# *Ex vivo* imaging of active caspase 3 by a FRET-based molecular probe demonstrates the cellular dynamics and localization of the protease in cerebellar granule cells and its regulation by the apoptosis-inhibiting protein survivin

**DOI:** 10.1186/s13024-016-0101-8

**Published:** 2016-04-28

**Authors:** Laura Lossi, Carolina Cocito, Silvia Alasia, Adalberto Merighi

**Affiliations:** University of Turin, Department of Veterinary Sciences, Largo Paolo Braccini 2, I-10095 Grugliasco, TO Italy

**Keywords:** Neurons, Caspase 3, Survivin, Apoptosis, FRET, Biolistic transfection, Cerebellum, Organotypic cultures, Live imaging, Confocal microscopy

## Abstract

**Background:**

Apoptosis takes place in naturally occurring neuronal death, but also in aging, neurodegenerative disorders, and traumatic brain injuries. Caspase 3 (Casp3) is the most important effector protease in apoptosis: being inactive inside the cell, it undergoes enzymatic cleavage and - hence - activation once the apoptotic cascade is triggered. Immunological techniques with antibodies against cleaved Casp3 (cCasp3) or assays with colorimetric/fluorogenic substrates are commonly in use, but they do not allow to directly follow the dynamics of activation in alive neurons that may be committed to die.

**Results:**

By combined biolistic transfection, confocal microscopy, and fluorescence resonance energy transfer (FRET), we have implemented a methodology to dynamically monitor Casp3 activation in organotypic cerebellar slices from postnatal mice. After transfection with pSCAT3 FRET probes, we measured the ratio of the emissions of the donor/acceptor pair (ECFP_em_/Venus_em_) in fixed or alive cultures. In so doing, we *i.* discriminated the cellular compartment(s) of enzyme activation (nucleus, perikaryon, neurites); *ii.* demonstrated that Casp3 was constitutively active in the granule cells; *iii.* followed the fluctuations of ECFP_em_/Venus_em_, and its response to 25 mM KCl depolarization, or to increased intracellular Ca^++^ after NMDA (1 mM), kainic acid (1 mM), or A23187 (100–200 μM). The specificity of the active pSCAT3-DEVD probe was confirmed with RNA interference and after inhibition of Casp3 with Ac-DEVD-CMK (100 μM), as both sets of experiments brought ECFP_em_/Venus_em_ to the values recorded with the control probe pSCAT3-DEVG. After double-transfection with pSCAT3-DEVD + pHcRed1-C1-survivin, we also showed a 44–56 % reduction of basal Casp3 activity in cells overexpressing survivin, a protein-member of the family of apoptosis inhibitors, with augmented survival (2.82 folds). Survivin-rescued cells were sensitive to 5 mM H_2_O_2_ oxidative stress but died without intervention of Casp3.

**Conclusions:**

This *ex vivo* FRET-based methodology provides quantitative information on the functional and histological dynamics of Casp3 activation in individual neurons at a cell level resolution. Not only it can be combined with experimental manipulation of the apoptotic machinery inside the cell, but offers several advantages over existing protocols for monitoring apoptosis in live mammalian neurons, and has potential to be transferred *in vivo*. Due to the pivotal role of Casp3 in apoptosis, our approach is relevant for a better comprehension of molecular neurodegeneration in the normal and pathological brain.

**Electronic supplementary material:**

The online version of this article (doi:10.1186/s13024-016-0101-8) contains supplementary material, which is available to authorized users.

## Background

Apoptosis is a well-known form of programmed cell death (PCD), the apoptotic program being triggered at genomic level and leading to specific biochemical and ultrastructural cellular alterations [1]. The term naturally occurring neuronal death (NOND) was coined to highlight the physiological role of PCD in the maturation of neurons and their connections [2]. However, apoptosis is also responsible for neurodegeneration and neuronal loss in aging, neurodegenerative disorders and traumatic brain injuries [1].

Caspases are a family of related proteases playing several important functions in apoptosis. They are essential to completion of PCD [3–5], and are activated in a cascade leading to rapid disablement of key cell structural proteins, chromatin condensation and DNA fragmentation, cell shrinkage and blebbing [6].

Caspase 3 (Casp3) is the most important executioner caspase [7, 8]: it is ubiquitous in inactive form, but becomes enzymatically cleaved in apoptotic cells that thus harbor the active protease (cleaved Casp3 - cCasp3) [9]. It is therefore not surprising that significant efforts have been devoted to the development of specific assays to monitor Casp3 activity in tissues and cells. Production of specific antibodies has been a major breakthrough [10], but immunocytochemistry (ICC), ELISA, or Western blotting, and assays with colorimetric or fluorogenic substrates do not allow a direct analysis of Casp3 activation dynamics during cell death and/or in response to cellular stressors. To overcome such a limitation, alternative approaches have been sought for. For example, in the past we have used the ApoAlert™ pcaspase3-sensor vector to analyze the cleavage of Casp3 in the course of cerebellar NOND [11]. This approach, however, was not amenable to quantitative studies, and thus of limited value for further pharmacological characterization. Likewise, others have used different types of functionalized probes for optical imaging of Casp3 in isolated neurons or in the intact brain and retina after experimentally-induced apoptosis [12–15].

The bulk of studies on Casp3 activation have been carried out *in vitro*, using primary neurons and/or neuronal cell lines. These approaches offer good opportunities to unravel the intervention of the protease in neuronal PDC, allowing to pharmacologically challenge homogeneous cell populations, and to easily investigate cause-to-effect correlations. However, they do not obviously permit analysis of the interplay between different types of neurons, or neurons and glia. Yet the study of NOND *in vivo* is challenging, and substantial difficulties need to be faced when tackling it. Most important are the asynchrony of the process, which, within the same brain area, affects several different types of neurons at different times; its close relationship with proliferation; and the very rapid clearance of apoptotic cells from tissue by the microglia [10]. Despite of these difficulties, observations in intact animals not only have shown that the cerebellar granule cells (CGCs) - the most abundant type of cortical neurons in cerebellum - undergo an apoptotic type of NOND shortly after their generation, but also that some underlying molecular mechanisms are different in CGC progenitors/precursors or fully differentiated CGCs [16].

Brain organotypic slices represent an optimal tool for analysis of NOND and experimentally-induced neuronal death *ex vivo*, as reviewed in [17]. We here describe a methodology to study Casp3 activation in organotypic cerebellar cultures (OCCs) by the combination of biolistic transfection, laser scanning confocal fluorescence microscopy (LSCFM), and fluorescence resonance energy transfer (FRET). LSCFM allows exciting small spatial volumes with submicron resolution, to provide different simultaneous readouts (intensity, spectral characteristics), and to measure fluorescence emissions from different channels in the same tissue volume [18]. Here used in combination with FRET, a technology that enables detecting protein-to-protein interactions in living cells [19], LSCM not only permitted the visualization of Casp3 activity in individual neurons, but also yielded quantitative information about the dynamics of protease activation. In addition, we implemented a double-transfection protocol to demonstrate the interplay between Casp3 and survivin, a critically required protein for survival of developing CNS neurons that also intervenes in neural repair, and neurodegeneration [20].

## Results and discussion

### Transfection of OCCs

**Fig. 1:**
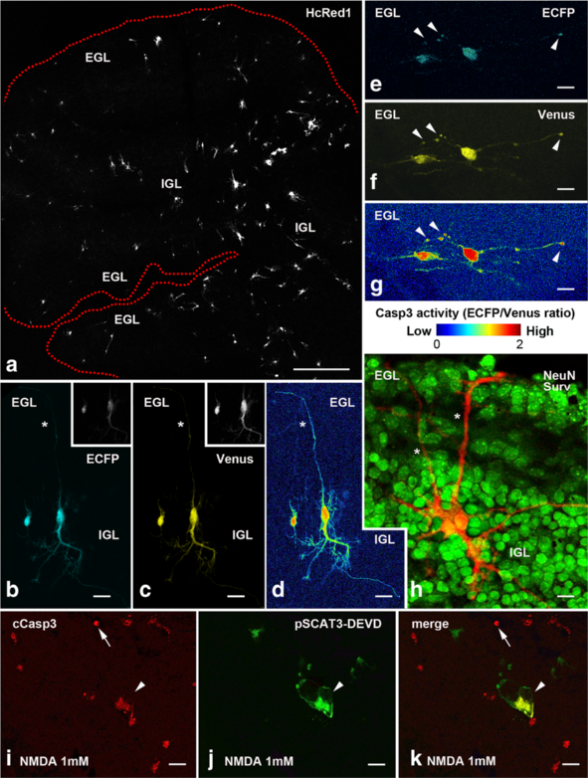
Visualization of Casp3 activation in fixed OCCs after biolistic transfection. **a** Low magnification image of a double-transfected OCC (pSCAT3-DEVD + pHcRed1-C1) after excitation with the 588 nm argon laser line. HcRed1 expression permits an easy visualization, localization, and identification of successfully transfected cells. The red-dotted line indicates the border of the culture. **b**-**g** Exemplificative images of two CGCs in the IGL (**b**-**d**) and two CGCs (**e**-**g**) in the EGL after pSCAT3-DEVD transfection showing the emissions of the FRET pair at 475 nm (ECFP) and 530 nm (Venus). The cell at right in **b**-**d** is a CGC in the vertical bipolar stage of migration and displays a well visible axon (asterisk) that bifurcates to give origin to a parallel fiber. The two cells in **e**-**g** are CGCs at the horizontal bipolar stage of migration. The cell at right displays some enlargements of its processes with high Casp3 activity (*arrowheads*). Note that to better show the distribution of ECFP and Venus images are taken at different laser excitation powers. As an example, the true fluorochrome emissions during FRET recording are shown in black and white in the inserts of panels **b** and **c**. In **d** and **g** cells are imaged in pseudocolor using a logarithmic scale to display the ECFP_em_/Venus_em_ ratio. Note the cellular resolution of the FRET probe. **h** Combined ICC for the marker NeuN (*green channel*) and biolistic transfection with pHcRed1-Surv (*red channel*) shows two transfected CGCs in the IGL. Both cells are in the vertical bipolar stage and their axons have been labeled by the *asterisks*. Image has been modified and reproduced with permission from [29]. **i**-**k** Combined ICC for cCasp3 (*red channel*) and biolistic transfection with pSCAT3-DEVD (*green channel*) after induction of apoptosis with 1 mM NMDA for 48 h shows several cCasp3 immunoreactive cells. The pattern of cellular localization of the 17/19 kDa fragment of the protease is different among cells, one of which (*arrow*) displays a highly condensed cCasp3 positive nucleus. The larger cell transfected with pSCAT3-DEVD displays cytoplasmic cCasp3 immunoreactivity, but the nucleus (*arrowhead*) is negative. *Abbreviations*: cCasp3 = cleaved caspase 3; CGC = cerebellar granule cell; ECFP = enhanced cyan fluorescent protein; EGL = external granular layer of forming cerebellar cortex; IGL = internal granular layer of forming cerebellar cortex; NeuN = nuclear nuclei antigen; NMDA = N-methyl-D-aspartate; Surv = survivin; Venus = mutated yellow fluorescent protein. Scale bars: A = 500 μm; b-k: 10 μm

According to the experimental need, OCCs were transfected with plasmids encoding any of the following fluorescent reporter proteins (FRPs): enhanced cyan fluorescent protein (ECFP) – cyan emission; green fluorescent protein (GFP) – green emission; the Venus variant of yellow fluorescent protein (YFP) – yellow emission; and wild-type Discosoma red fluorescent protein (DsRed1), humanized (HcRed1) – red emission. Starting from twenty-four hours post-transfection (HPT), fluorescent cells were easily spotted in OCCs with appropriate filter combinations (wide-field fluorescence microscopy) or excitation/emission settings (LSCFM). The peak of FRP expression was achieved around forty-eight to seventy-two HPT. Subsequently, neither there was an obvious increase/reduction of fluorescence intensity in individual cells, nor in the number of fluorescent cells, but FRPs were still detectable in cultures maintained *in vitro* up to two weeks. Although numbers were variable, from several tens up to a few hundreds of successfully transfected neurons/slice were yielded after a single Gene Gun® shot (Fig. 1a).

Biolistic transfection of murine OCCs with plasmid cDNAs using the human cytomegalovirus (hCMV) as a promoter, for the most tagged the CGCs [21]. In keeping with these previous observations, successfully transfected neurons were easily identified as post-mitotic CGCs in the course of axonogenesis [22] on the basis of their morphologies (Fig. 1b-g), mean size (see also Specificity of the pSCAT3 probe for Casp3), and after combined ICC with the specific marker NeuN [23] (Fig. 1h). We carried out all FRET measurements on these cells at 48 HPT (fixed tissue) or from then on (live imaging). The rationale and results of experiments are summarized in Table 1.

**Table 1:** List of experiments, their rationale and main results

Exp #	Culture conditions	Transfection protocol	Treatment(s)	Experiment rationale
				Main results
1	• Medium 1 (horse serum) – 4 DIV • Transfection • Medium 1 (horse serum) – 2 DIV • FRET on fixed OCCs	Single pcDNA-SCAT3 DEVG (FRET control probe)	None	• Assessment of theoretical probe efficiency by acceptor photobleaching • Exp #2 control: confirmation that cellular caspase 3 cannot cleave the mutated DEVG sequence
				• FRET_eff_ can be measured in OCCs
2		Single pcDNA-SCAT3 DEVD (FRET probe)	None	• Calculation of ECFP_em_/Venus_em_ in serum-containing medium to measure basal caspase 3 activation
				• Caspase 3 is constitutively active in CGCs • Presence of serum reduces basal ECFP_em_/Venus_em_ (means that caspase 3 activity is reduced)
3	• Medium 1 (horse serum) – 4 DIV • Transfection • Medium 2 (Neurobasal) – 2 DIV • FRET on fixed OCCs	Single pcDNA-SCAT3 DEVG (FRET control probe)	None	• Assessment of theoretical probe efficiency by acceptor photobleaching • Exp #4 control: confirmation that cellular caspase 3 cannot cleave the mutated DEVG sequence
				• FRET_eff_ can be measured in OCCs
4		Single pcDNA-SCAT3 DEVD (FRET probe)	None	• Assessment of ECFP_em_/Venus_em_ in serum-free medium to measure basal caspase 3 activation
				• Caspase 3 is constitutively active in CGCs
5	• Medium 1 (horse serum) – 4 DIV • Transfection • Medium 1 (horse serum) – 2 DIV • FRET on fixed OCCs	Double pcDNA-SCAT3 DEVG (FRET control probe) + Casp3 shRNA control clone	None	• Negative control for caspase 3 RNAi
				• ECFP_em_/Venus_em_ after transfection with FRET control probe is not modified by co-transfected control shRNA clone
6		Multiple pcDNA-SCAT3 DEVG (FRET control probe) + Casp3 shRNA clones 1–4	None	• Negative control for caspase 3 RNAi
				• ECFP_em_/Venus_em_ after transfection with FRET control probe is not modified by co-transfected Casp3 shRNA clones
7	• Medium 1 (horse serum) – 4 DIV • Transfection • Medium 1 (horse serum) – 2 DIV • FRET on fixed OCCs	Double pcDNA-SCAT3 DEVD (FRET probe) + Casp3 shRNA control clone	None	• Negative control for caspase 3 RNAi
				• ECFP_em_/Venus_em_ after transfection with FRET probe is not modified by co-transfected control shRNA clone
8		Multiple pcDNA-SCAT3 DEVD (FRET probe) + Casp3 shRNA clones 1–4	None	• Demonstration of probe specificity by caspase 3 RNAi
				• ECFP_em_/Venus_em_ after transfection with FRET probe is reduced by co-transfected Casp3 shRNA clones • Constitutively active caspase 3 can be effectively silenced by RNAi
9	• Medium 1 (horse serum) – 4 DIV • Transfection • Medium 1 (horse serum) – 2 DIV • FRET on fixed OCCs	Single pcDNA-SCAT3 DEVG (FRET control probe)	Ac-DEVD-CMK 100 μM	• Negative control for caspase 3 inhibition
				• ECFP_em_/Venus_em_ after transfection with FRET control probe is not modified in the presence of caspase 3 inhibitor
10		Single pcDNA-SCAT3 DEVD (FRET probe)	Ac-DEVD-CMK 100 μM	• Demonstration of probe specificity by specific inhibition of caspase 3
				• ECFP_em_/Venus_em_ after transfection with FRET probe is reduced by caspase 3 inhibitor • Constitutively active caspase 3 can be effectively inhibited by Ac-DEVD-CMK
11	• Medium 1 (horse serum) – 4 DIV • Transfection • Medium 2 (Neurobasal) – 2 DIV • FRET on fixed OCCs	Double pcDNA-SCAT3 DEVD (FRET probe) + pHcRed1-C1	None	• Method control: a) Evaluation of co-transfection efficiency; b) Evaluation of possible interferences of Red1-C1 fluorescence with FRET • Further morphological identification of transfected cells • Exp #12 control
				• a) Co-transfection rate is 100 %; b) Red1-C1 does not interfere with FRET • Confirmation of Exp #3: caspase 3 is constitutively active in CGCs • Transfected cells are for the most CGCs
12		Double pcDNA-SCAT3 DEVD (FRET probe) + pHcRed1-C1-Surv	None	• Evaluation of the effects of survivin overexpression on basal levels of caspase 3 activation • Evaluation of the effect of survivin on cell survival
				• Survivin reduces basal caspase 3 activity • Survivin promotes cell survival
13	• Medium 1 (horse serum) – 4 DIV •Transfection • Medium 1 (horse serum) – 2 DIV • FRET on fixed OCCs	Double pcDNA-SCAT3 DEVD (FRET probe) + pHcRed1-C1	None	• Method control: a) Evaluation of co-transfection efficiency; b) Evaluation of possible interferences of Red1-C1 fluorescence with FRET • Further morphological identification of transfected cells • Exp. #14 control
				• a) Co-transfection rate is 100 %; b) Red1-C1 does not interfere with FRET • Confirmation of Exp #4: caspase 3 is constitutively active in CGCs • Transfected cells are for the most CGCs
14		Double pcDNA-SCAT3 DEVD (FRET probe) + pHcRed1-C1-Surv	None	• Evaluation of the effects of survivin overexpression on basal levels of caspase 3 activation • Evaluation of the effect of survivin on cell survival
				• Survivin reduces caspase 3 activity • Survivin promotes cell survival
15	• Medium 1 (horse serum) – 4 DIV • Transfection • Medium 2 (Neurobasal) – 2 DIV • FRET on fixed OCCs	Single pcDNA-SCAT3 DEVG (control probe) or pcDNA-SCAT3 DEVD (FRET probe)	• 25 mM KCl • 1 mM NMDA • 1 mM KA • 100–200 μM A23187 • 50 μM–25 mM H_2_O_2_	• Measurement of caspase 3 activity after induction of apoptosis
				• A subpopulation of CGCs is insensitive to apoptosis induction and does not display changes in ECFP_em_/Venus_em_ • Other cells display signs of sufferance
16	• Medium 1 (horse serum) – 4 DIV • Transfection • Medium 2 (Neurobasal) – 1 DIV • FRET on alive OCCs	Single pcDNA-SCAT3 DEVD (FRET probe)	• 60 mM KCl • 100 mM H_2_O_2_	• Real time monitoring of caspase 3 in basal conditions • Measurement of caspase 3 activity after depolarization or oxidative stress
				• Caspase 3 activity can be measured in real time experiments • Increase of ECFP_em_/Venus_em_ after K^+^ depolarization but not cell death • Only tendency to increase of ECFP_em_/Venus_em_ after oxidative stress. Reduction in the number of transfected cells

### Cellular resolution of the pSCAT3 probe

In previous studies with pSCAT3, it was possible to exploit the quantitative nature of FRET for obtaining information about the site(s) of cellular localization of cCasp3. This was done by expressing the ratio of the emissions of the two FRET fluorophores in a pseudocolor scale [24]. We have used here a similar approach and a logarithmic pseudocolor RGB scale to express the value of ECFP_em_/Venus_em_ (Fig. 1d and g). Using this scale, the cellular regions where Casp3 activity was high appeared in red, whereas areas of low activity were blue. Figure 1d and G are exemplificative pseudocolor images of four CGCs with an intact morphology and different levels of activation of Casp3. Notably, in all the four cells the nucleus displayed high Casp3 activity, but there were also spots of intense activation within the cellular processes in one of these cells (Fig. 1g). These observations were in full accord with the demonstration that, after proteolytic activation and the recognition of its substrate protein(s), Casp3 translocates into the nucleus to trigger cellular demolition [25]. The level of resolution of the probe was so precise that also individual axons could be imaged, and the well-known different phases of CGC axonogenesis [22] were easily recognized.

By the use of ICC and a specific antibody directed against cCasp3, we have previously shown in mouse [11] and rabbit [16] that, once activated, the enzyme can localize to both the cytoplasm and the nucleus of CGCs. However, the low resolution of enzyme ICC did not allow to easily discriminating the cellular compartment of Casp3 localization: precipitation of 3, 3′ diaminobenzidine into the cytoplasm often completely obscured the nucleus, impeding its proper observation [16], and only slight improvements were achieved by immunofluorescence and combined DAPI nuclear staining [11]. Yet, this information was merely qualitative and a correct interpretation of results remained difficult.

### FRET efficiency (FRET_eff_) of the pSCAT3 probe and accuracy of FRET measurements in OCCs

For FRET to occur, a pair formed by a donor and an acceptor molecule is required. The donor fluorescent molecule is then excited and, if the acceptor molecule is close enough to it, part of its emitted fluorescent light is transferred to the acceptor. Thus, FRET has become increasingly important in cell biology for its ability to measure the distance between molecules on a scale of a few nanometers that is far below the resolution of optical fluorescence microscopes [26]. However, to correctly interpret and validate FRET experiments it is important to first establish their degree of accuracy.

In the pSCAT3 FRET probes, the donor (ECFP) and the acceptor (Venus) are genetically engineered into the cell. The original studies conducted with these probes have well depicted the space-time dynamics of the activation of Casp3 in isolated cells [24]. Subsequent studies analyzed the time course of apoptosis in different organs of a relatively simple organism such is *Drosophila* [27, 28]. Imaging the more complex mammalian nervous system in organotypic cultures poses additional problems [29]. Therefore, we first established whether the pSCAT3 vector was effectively transfected and functional in OCCs.

**Fig. 2:**
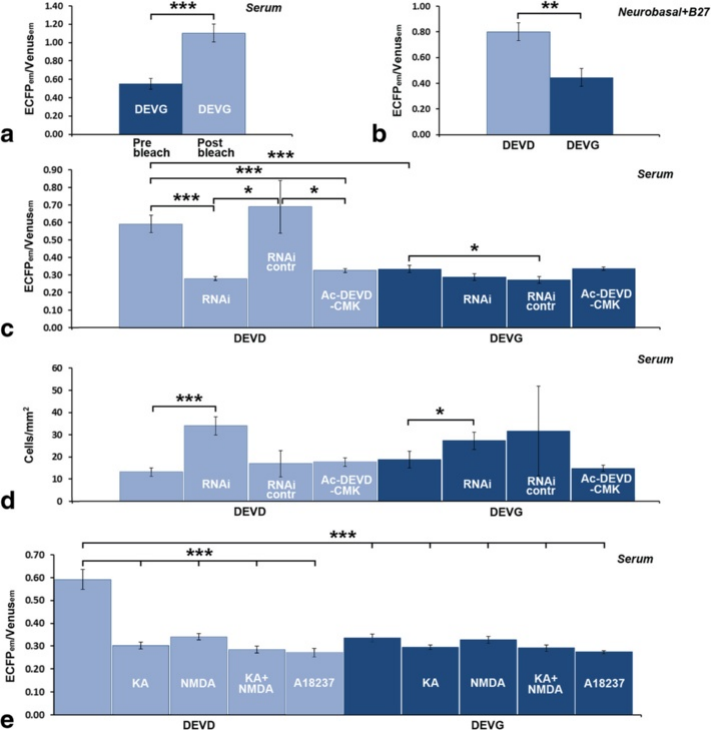
FRET measurements of Casp3 activity in fixed OCCs and specificity controls. **a** calculation of FRET_eff_ of the SCAT3 probes by acceptor photobleaching (experiment #3). There is a statistically significant difference in the ECFP_em_/Venus_em_ ratio before and after photobleaching of pSCAT3-DEVG. The mean value of FRET_eff_ was 0.22 ± 0.02 (see text and Additional file 1 for further information). **b** A comparison of the ECFP_em_/Venus_em_ mean values after transfection with pSCAT3-DEVD (FRET probe) or pSCAT3-DEVG (control probe) shows a reduction of about 56 % in ECFP_em_/Venus_em_ in cells transfected with the control probe. As in the control probe cellular Casp3 cannot cleave the FRET pair, this experiment demonstrates that the enzyme is constitutively active in CGCs in the absence of any pharmacological treatment. After transfection, OCCs were maintained in medium 2, containing Neurobasal and B27 supplement. **c** Specificity of the pSCAT3-DEVD probe for Casp3 is demonstrated after RNAi and Ac-DEVD-CMK inhibition. After multiple transfection with pSCAT3-DEVD and the mix of the four shRNA plasmids targeting the Casp3 gene or with pSCAT3-DEVD in the presence of the caspase inhibitor Ac-DEVD-CMK (100 μM) there is a significant reduction in the mean value of ECFP_em_/Venus_em_ in comparison to that measured from OCCs transfected with pSCAT3-DEVD alone (pastel blue bars). There are notably no differences in mean values of ECFP_em_/Venus_em_ when the pSCAT3-DEVG control probe was employed (blue bars), with the exception of OCCs transfected with a combination of pSCAT3-DEVG and the RNAi control clone [pSCAT3-DEVG 0.33 ± 0.01 (294 cells); pSCAT3-DEVG + RNAi control 0.27 ± 0.02 (100 cells); *P* = 0.01]. This result is somewhat puzzling as pSCAT3-DEVG is insensitive to Casp3 cleavage. It is also worth noting that after co-transfection with pSCAT3-DEVD + RNAi control values of ECFP_em_/Venus_em_ displayed higher variance (0.21) and, consequently, SEM was much higher (0.15 – see bars in graph) than in all other tested conditions. To explain these data we can only speculate that transfection with a Casp3-unrelated RNAi control clone of unknown specificity (such as that provided by manufacturer) may somehow interfere with protein synthesis machinery in cells. **d** TCD after RNAi and Ac-DEVD-CMK inhibition of Casp3. There is a statistically significant increase of TCD after multiple transfection with shRNA plasmids that epigenetically inhibit the casp3 gene together with pSCAT3-DEVD (pSCAT3-DEVD 13.13 ± 1.80, n. cells = 584; pSCAT3-DEVD + RNAi 33.89 ± 4.09, n. cells = 840; *P* = 0.000926) or pSCAT3-DEVG (pSCAT3-DEVG 18.84 ± 3.72, n. cells = 941; pSCAT3-DEVG + RNAi 27.37 ± 3.91, n. cells = 1391; *P* = 0.04279), but not in RNAi control experiments and after Ac-DEVD-CMK [n. cells = 213 (pSCAT3-DEVD + RNAiCONTR), 585 (pSCAT3-DEVD+ Ac-DEVD-CMK), 133 (pSCAT3-DEVG + RNAiCONTR), 794 (pSCAT3-DEVG+ Ac-DEVD-CMK)]. In these experiments, we have counted a total of 5,841 transfected cells and measured their areas. Mean area was 165.71 ± 2.16 μ^2^, corresponding to a mean diameter of 15 μm for a circular object. **e** Transfection with pSCAT3-DEVD or pSCAT3-DEVG demonstrates the existence of a subpopulation of CGCs resistant to induction of apoptosis by ionotropic glutamate receptor agonists and A23187 after measurement of mean values of ECFP_em_/Venus_em_ (pSCAT3-DEVD 0.59 ± 0.04; n. cells = 251; pSCAT3-DEVD + KA 0.30 ± 0.01; n. cells = 103; pSCAT3-DEVD + NMDA 0.34 ± 0.01; n. cells = 105; pSCAT3-DEVD + KA + NMDA 0.28 ± 0.01; n. cells = 62; pSCAT3-DEVD + A23187 0.27 ± 0.01; n. cells = 132; pSCAT3-DEVG 0.33 ± 0.01 n. cells = 294; pSCAT3-DEVG + KA 0.29 ± 0.01; n. cells = 98; pSCAT3-DEVG + NMDA 0.33 ± 0.01; n. cells = 80; pSCAT3-DEVG + KA + NMDA 0.29 ± 0.01; n. cells = 68; pSCAT3-DEVG + A23187 0.27 ± 0.005; n. cells = 7). Error bars = SEM. *P-value 0.05–0.01 ** *P*-value < 0.01–0.001 % *** *P*-value < 0.001 %

FRET efficiency (FRET_eff_) is commonly used to assess the functionality of a FRET probe inside living cells [30]. In a preliminary set of experiments, we have calculated the theoretical range of variability of FRET_eff_ in OCCs, to establish a dynamic range of work for a correct interpretation of subsequent studies. Due to its molecular nature, the functional FRET probe (pSCAT3-DEVD) is sensitive to any Casp3 active inside the cell that cleaves its consensus sequence DEVD, impeding FRET to occur. Therefore, calibrating experiments were carried out with the control probe pSCAT3-DEVG, which is insensitive to Casp3 activity as donor and acceptor cannot be separated by the proteolytic activity of the protease (Table 1: experiments #1 and 3): after transfection, fluorescence emissions of the donor (ECFP_em_) and of the acceptor (Venus_em_) were measured *before* and *after* bleaching of the acceptor. FRET_eff_ was then calculated as indicated in Methods and in Additional file 1. The ratio of ECFP_em_ and Venus_em_ (ECFP_em_/Venus_em_ - in gray scale) was significantly increased after bleaching (Fig. 2a and Additional file 1), with a statistically significant variation from 0.55 ± 0.06 (pre-bleach) to 1.10 ± 0.10 (post-bleach) (n. cells =12, t-Test: *P* = 0.0001). Remarkably, it was calculated that the ECFP_em_/Venus_em_ normalized value changed from 0.43 to 1.11 in HeLa cells transfected with pSCAT3-DEVD and challenged with tumor necrosis factor α-cycloheximide, a strong activator of Casp3 [24]. Therefore, acceptor photobleaching experiments confirmed that the probe was sufficiently transcribed also into an organotypic context and herein worked with comparable dynamic range to that observed in isolated cells, as the pre-bleaching condition mimicked a healthy cell in which Casp3 was inactive, whereas the post-bleaching condition simulated an apoptotic cell where cCasp3 cleaved the ECFP/Venus pair.

The mean value of FRET_eff_ in our experiments was 0.22 ± 0.02 (Additional file 1). In discussing this figure, one should consider how FRET_eff_ was calculated [31, 32] and the type and nature of the FRET probe. For the sake of brevity, we have discussed these two issues in Additional file 2 where we also provided quantitative data and relevant references for comparative purposes.

### Casp3 is constitutively active in CGCs and activity is not significantly affected by culture conditions

In experiments #1–4 we have measured ECFP_em_/Venus_em_ in CGCs transfected with pSCAT3-DEVG (control) or pSCAT3-DEVD in the absence or presence of serum in culture medium. We wanted to establish whether or not Casp3 was active in our OCCs and if serum had an effect - if any - on the level of Casp3 activity. The mean ratio of the emissions of two fluorophores after pSCAT3-DEVD transfection was significantly higher of that recorded in cells transfected with pSCAT3-DEVG independent from serum [serum-free medium: pSCAT3-DEVD 0.80 ± 0.07; n. cells = 10 and pSCAT3-DEVG 0.45 ± 0.07; n. cells = 8; t-Test: *P* = 0.003 (Fig. 2b); serum: pSCAT3-DEVD 0.68 ± 0.02; n. cells = 251 and pSCAT3-DEVG 0.33 ± 0.01; n. cells = 294; t-Test: *P* = 5.37974E-31]. Notably, the absence/presence of serum did not significantly interfere with the mean of readouts of ECFP_em_/Venus_em_ after transfection with pSCAT3-DEVD (serum-free medium: 0.80 ± 0.07 n. cells = 10 and serum-containing medium: 0.68 ± 0.02; n. cells = 251; t-Test *P* = 0.15) or pSCAT3-DEVG (serum-free medium: 0.35 ± 0.001, n. cells = 65) and serum-containing medium: 0.29 ± 0.005, n. cells = 79); t-Test *P* = 0.25]. Therefore, even when OCCs were not subjected to any experimental challenge, Casp3 was constitutively active in individual CGCs (see also experiments #5–7 and 9 that provided controls for RNAi and Ac-DEVD-CMK inhibition of Casp3).

Using the Caspase-3 sensor™ probe, our previous studies in murine OCCs have been successful in showing the activation of Casp3 in CGCs [11]. The probe consisted of a fluorescent tag (EYFP) that translocated from the cytoplasm - when Casp3 was inactive - to the cell nucleus, once the enzyme was activated. However, quantitative studies were made possible only in terms of counting tagged cells with different patterns of subcellular EYFP distribution (nucleus/cytoplasm fluorescence), and it was impossible to quantitate Casp3 activation. Instead, our present observations provide a quantitative readout of Casp3 basal activation in NOND, and are in full agreement with data reported for isolated CGCs using the QCASP3.2 probe [15].

### Specificity of the pSCAT3 probe for Casp3

Once established that FRET probes were technically suited for slice applications and detected basal levels of the protease in OCCs, a key issue remained to be addresses, *i.e.* the specificity of the method for Casp3, as one should not omit mentioning that the DEVD consensus sequence can be also recognized by Casp7 [33, 34]. To fully clarify this issue we have devised two sets of experiments (Table 1, experiments #5–10 and Fig. 2c-d) using either short-hairpin RNAs (shRNAs) to knockdown Casp3 by means of RNA interference or Ac-DEVD-CMK, a widely-employed inhibitor of the protease [35].

In the first set of experiments, RNAi was performed by taking advantage of one of the most appealing features of biolistic transfection, *i.e.* the possibility to simultaneously transfect multiple cDNAs inside the cell at known stoichiometric ratios [28]. In these experiments, we have measured ECFP_em_/Venus_em_ in CGCs transfected with pSCAT3-DEVG or pSCAT3-DEVD together with a control shRNA or a mix of four different shRNAs targeting the Casp3 gene (see also figure in Additional file 3). In the second set of experiments we have instead measured ECFP_em_/Venus_em_ in CGCs transfected with pSCAT3-DEVG or pSCAT3-DEVD in the presence of 100 μM Ac-DEVD-CMK. The mean ratio of the emissions of two fluorophores was significantly lower when slices were transfected with pSCAT3-DEVD + Casp3 shRNAs [pSCAT3-DEVD 0.59 ± 0.04; n. cells = 251 and pSCAT3-DEVD + Casp3 shRNAs 0.28 ± 0.02; n. cells = 102); t-Test: *P* = 2.27714E-08]. A statistically significant reduction of ECFP_em_/Venus_em_ was also observed after transfection with pSCAT3-DEVD in the presence of the inhibitor (pSCAT3-DEVD 0.59 ± 0.04; n. cells = 251 and pSCAT3-DEVD+ Ac-DEVD-CMK 0.32 ± 0.01; n. cells = 104; t-Test: *P* = 8.62803E-07). On the other hand, the mean value of ECFP_em_/Venus_em_ was unchanged in experiments after transfection with pSCAT3-DEVD + control shRNA (pSCAT3-DEVD 0.59 ± 0.04; n. cells = 251 and pSCAT3-DEVD + control shRNA 0.69 ± 0.15; n. cells = 106; t-Test: *P* = 0.55), or in RNAi and Ac-DEVD-CMK experiments performed with the Casp3-insensitive probe pSCAT3-DEVG (Fig. 2c blue bars).

These experiments unequivocally proved the specificity of the FRET probe for Casp3, but, as a further validation, we also decided to calculate the density of transfected cells (TCD) and value of ECFP_em_/Venus_em_ after excitation of ECFP with a laser line at 405 nm.

As to TCD, we reasoned that successful Casp3 RNAi should match to improved cell survival, the constitutive activity of the protease being nullified by post-transcriptional gene silencing. In keeping with this assumption, we observed that, after RNAi, TCD was increased 2.58 fold in experiments using pSCAT3-DEVD (Fig. 2d pastel blue bars), and 1.45 fold with pSCAT3-DEVG (Fig. 2d blue bars). Notably, the graph in Fig. 2d also shows that transfection with the RNAi control clone, as well as treatment with the inhibitor Ac-DEVD-CMK yielded values of TCD not statistically different from those after any of the two pSCAT3 probes alone. That Ac-DEVD-CMK had no statistically significant consequences on TCD was likely due to the fact that the inhibitor was not specifically targeted to the transfected cells. We did not calculate the *total* density of cells in OCCs, but it is well possible that an increase occurred in the presence of Ac-DEVD-CMK. Nonetheless, an increase, if any, had not measurable consequence on transfection efficiency, a fact that further validates the use of biolistics in quantitative studies of PCD.

The use of extraction procedures was not practicable to validate RNAi experiments. Thus, obtaining unequivocal proof for successful transfection with shRNAs required to employ plasmids that also encoded for GFP. In this way, we checked for GFP expression together with ECFP/Venus in individual cells after biolistics (see figure in Additional file 3). To exclude artifacts due to fluorescence bleed-through between ECFP and GFP, we also have measured FRET after excitation of ECFP at 405 nm. The results of these experiments are reported in Additional file 3 and show that expression of GFP in transfected cells was not detrimental to successful recording of FRET.

Collectively, all experiments on probe validation converge to demonstrate the specificity of pSCAT3-DEVD for Casp3. It was initially demonstrated that Casp7, which is also able to cleave the DEVD tetrapeptide, was present at very low levels or undetectable in the brain [25]. However, we previously shown, by ICC and Western blotting, that a few cells in the internal granular layer (IGL) of the forming rabbit cerebellar cortex *in vivo* were immunoreactive for cCasp7 but, surprisingly, not for the uncleaved pro-caspase [16]. These results were puzzling and raised some doubts on the specificity of the primary antibodies there employed. In keeping with the possibility that a false positive reaction was occurring in our rabbit material, a very recent study has excluded that Casp7 cleaved the tetrapeptide DEVD in primary cultured rat CGCs [15]. It should also be recalled here that cCasp7, differently from cCasp3, does not undergo nuclear translocation [33]. Thus, besides to RNAi and Ac-DEVD-CMK experiments, a further proof for the pSCAT3-DEVD specific recognition of Casp3 came from subcellular distribution imaging, and led to safely conclude that pSCAT3-DEVD was specifically detecting Casp3 and not Casp7 activity in organotypically cultured CGCs.

### Experimental induction of apoptosis demonstrates that Casp3 does not mediate cell death in a subpopulation of CGCs

**Fig. 3:**
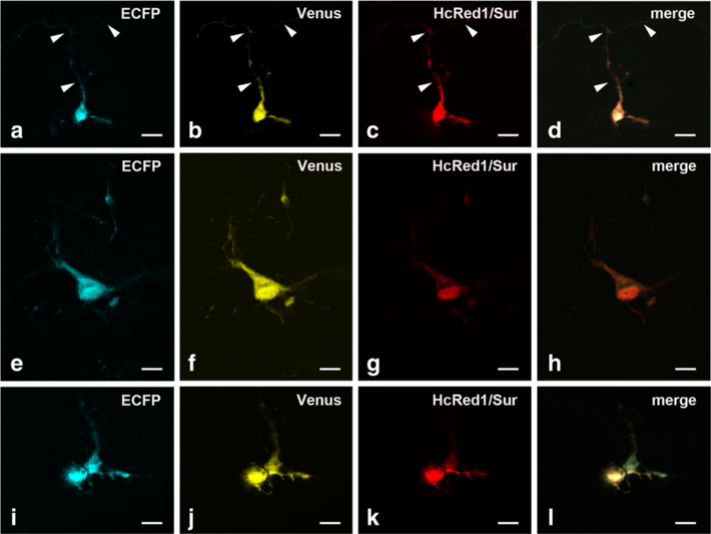
Exemplificative images of the different morphologies of pSCAT3-DEVD and pHcRed1-C1-survivin double-transfected cells. **a**-**d** A CGC with the typical vertical bipolar stage morphology in the IGL. The arrow-heads indicate the axon and its branching into the parallel fiber. **e**-**h** A presumptive Golgi cell in the IGL can be identified as being larger and displaying a multipolar morphology. **i**-**l** Two CGCs in the IGL display the typical features of early apoptosis: retraction of processes and a tendency to cell shrinkage. Note that to better show the distribution of ECFP and Venus as well as the cell morphology, images are taken at different laser excitation powers*. Abbreviations*: CGC = cerebellar granule cell; ECFP = enhanced cyan fluorescent protein; IGL = internal granular layer of forming cerebellar cortex; Surv = survivin. Scale bars: 10 μm

Changes in ionic concentrations inside the cell and/or in the extracellular milieu modulate apoptosis. The concentration of intracellular Ca^++^ ([Ca^++^]_i_), in particular, is crucial to trigger death, and, according to the so called “Ca^++^ set-point hypothesis”, [Ca^++^]_i_ is dynamically set to values optimal for neuronal survival [36]. Numerous molecules are able to increase [Ca^++^]_i_ by different mechanisms. Therefore, we evaluated the effects of some of these molecules on Casp3 activity. In experiment #15 (Table 1), OCCs were incubated under depolarizing conditions with 25 mM KCl, or with N-methyl-D-aspartate (NMDA - 1 mM), kainic acid (KA - 1 mM), NMDA+ KA (1 mM +1 mM) or A23187 (100 μM/24 h, or 200 μM/24 or 48 h). KCl was used at 25 mM, as this extracellular K^+^ concentration [K^+^]_e_ was found to be optimal for neuroprotection in OCCs [21]. The concentrations employed for glutamate receptors’ agonists and A23187 were by far exceeding those capable to induce apoptosis, as no differences in values of ECFP_em_/Venus_em_ were observed at lower concentrations (data not shown). We observed morphological signs of sufferance in OCCs after these latter treatments (Fig. 3i-l). Nonetheless, after apoptosis induction we did not observe statistically significant increases in the mean value of ECFP_em_/Venus_em_ in comparison to controls (Fig. 2e).

To further demonstrate that Casp3 was activated in OCCs challenged with pro-apoptotic glutamate agonists, we combined transfection with pSCAT3-DEVD with the subsequent ICC localization of cCasp3, using a specific antibody against the large fragment (17/19 kDa) of the activated protease. Figure 1i-k is exemplificative of the pattern of expression of cCasp3 following treatment with 1 mM NMDA, a pattern that is consistent with the changes in subcellular distribution of the enzyme that follow cleavage (see Cellular resolution of the pSCAT3 probe).

Similarly to what we have observed for Ac-DEVD-CMK (see Specificity of the pSCAT3 probe for Casp3), pro-apoptotic treatments had no statistically significant effects on the mean values of TCD in comparison to controls, in which OCCs were transfected with pSCAT3-DEVD or pSCAT3-DEVG and maintained in plain medium (data not shown). As we have also observed that maintenance in medium 1 in the presence of 60 mM KCl after transfection resulted in a massive cell death, with no fluorescent cells in OCCs, we believe that experiments described in this section prove, beyond any doubt, the existence of a subpopulation CGCs which is resistant to Casp3-mediated cell death. We exclude that the activity of Casp3 recorded by FRET is a technical artifact consequent to the preparation of brain slices, as we have previously demonstrated that, although apoptosis of some cerebellar cells indeed occurs immediately after explant, the latter equilibrates to the situation *in vivo* after a few DIV (specifically see Fig. 3b in [17]), and this is the reason why, in all experiments, OCCs were left to equilibrate for 4 DIV before transfection.

It was not surprising that 25 mM KCl did not affect the basal levels of activation of Casp3, considering that, as mentioned, at this [K^+^]_e_, KCl increases [Ca^++^]_i_ but acts as a pro-survival factor in OCCs (see [21] for further discussion). Conversely, we did not anticipate observing that some CGCs were resistant to ionotropic glutamate receptor agonists and A23187, a mobile ion-carrier that forms stable complexes with Ca^++^ ions and thereby increases [Ca^++^]_i_. NMDA and KA are, in fact, involved in glutamate excitotoxicity that follows a massive influx of Ca^++^ into the cell, ultimately leading to apoptosis [37]. Similarly, A23187 (calcimycin) acts as a pro-apoptotic factor and was used to modulate the [Ca^++^]_i_ and, hence, apoptosis in hippocampal slices subjected to oxygen/glucose deprivation [38].

In brief, this set of observations demonstrates that a subpopulation of CGCs was resistant to apoptosis induced by any of these three chemicals alone or in combination. In keeping with this possibility, 500 μM KA only slightly increased Casp3 activity in cultured CGCs, and neither Z-VAD.fmk, a pan-caspase inhibitor, nor the more specific Casp3 inhibitor, Ac-DEVD-CHO, prevented KA-induced cell death [39].

### Survivin overexpression reduces the basal levels of Casp3 activation in CGCs

To additionally confirm that Casp3 was active in CGCs and that its level of activation could be dynamically regulated, we have investigated the effects of survivin overexpression in these neurons. Survivin belongs to the family of inhibitors of apoptosis proteins (IAPs). It intervenes in the development and survival of different cellular types in virtually all tissues during embryogenesis, by taking part into mitosis and apoptosis [40]. It has been demonstrated that, after being translocated to the nucleus, survivin indirectly interacts with Casp3 to stop the apoptotic process [41]. The interaction with Casp3 takes place by means of a series of proteins located at different steps along the apoptotic cascade [42]. Therefore, we were confident that the pSCAT3-DEVD probe would allow detecting the effect of such an interaction and be valuable in monitoring the cellular response to a perturbation of the balance between pro-apoptotic factors and IAPs.

To study the effects of survivin overexpression onto the level of activity of Casp3 in individual CGCs, we first have double-transfected OCCs with pSCAT3-DEVD + pHcRed1-C1 to verify co-transfection efficiency, and to evaluate possible interferences of HcRed1 fluorescence with FRET measurements. We scanned OCCs under the LSCFM, and we observed a 100 % co-expression (>500 cells) of the three FRPs encoded by the two plasmid DNAs. Exemplificative images of double-transfected cells are reported in Fig. 3. Notably, HcRed1 did not interfere with the possibility to measure FRET (see Additional file 4), and, fortunately, it was uniformly distributed inside the cell to well depict the fine morphology of transfected neurons.

**Fig. 4:**
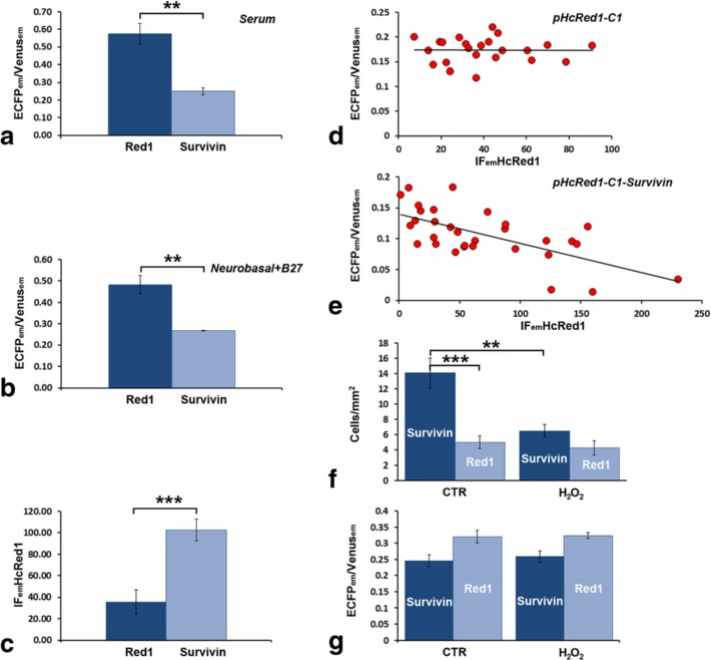
**a**-**b** Effect of survivin overexpression on the ECFP_em_/Venus_em_ values in OCCs cultivated in serum-containing medium (**a**) or Neurobasal + B27 supplement (**b**). In both culture conditions, survivin overexpression significantly reduces the constitutive activity of Casp3 in CGCs. **c** Survivin overexpression results in a statistically significant increase in the intensity of 618 nm fluorescence emission (in arbitrary units) of HcRed1 (IF_em_HcRed1) in CGCs transfected with pHcRed1-C1 or pHcRed1-C1-survivin. **d**-**e** Correlation between the intensity of 618 nm fluorescence emission (in arbitrary units) of HcRed1 (IF_em_HcRed1) and ECFP_em_/Venus_em_ in CGCs double transfected with pSCAT3-DEVD and pHcRed1-C1 (**d**) or pSCAT3-DEVD and pHcRed1-C1-survivin (**e**). Each dot represents a transfected cell. Note that IF_em_HCRed1is not correlated with Casp3 activity in cells transfected with the survivin control vector (**d**), whereas IF_em_HCRed1, and hence survivin level inside the cell (see text), is inversely correlated to Casp3 activity when CGCs are engineered to overexpress survivin (**e**). **f-g** Effect of H_2_O_2_ oxidative stress (5 mM) on the density of CGCs (**f**) or on the ECFP_em_/Venus_em_ value (**g**) after double transfection with pSCAT3-DEVD and pHcRed1-C1, or pSCAT3-DEVD and pHcRed1-C1-survivin. Note that oxidative stress has no effect on either the number/area of transfected cells or the ECFP_em_/Venus_em_ values when the cellular levels of survivin are not genetically manipulated. Survivin overexpression results in a statistically significant increase in cell density, and reduced, albeit not significantly, the ECFP_em_/Venus_em_ values after H_2_O_2_. Error bars = SEM. ** *P*-value 0.01–0.001 % *** *P*-value < 0.001 %

Then, we have compared the values of ECFP_em_/Venus_em_ in cells transfected with pSCAT3-DEVD + pHcRed1-C1 with those measured from cells transfected with pSCAT3-DEVD + pHcRed1-C1-survivin in the presence or absence of serum (Table 1 experiments #11–14). Figure 4a-b shows the effect of survivin overexpression on the mean values of ECFP_em_/Venus_em_ in the presence or absence of serum. Remarkably, in both conditions survivin overexpression significantly decreased these values (with serum - pSCAT3-DEVD + pHcRed1-C1: 0.57 ± 0.06, n. cells = 110; pSCAT3-DEVD + pHcRed1-C1-survivin: 0.25 ± 0.02, n. cells = 133; t-Test *P* = 0.005; without serum - pSCAT3-DEVD + pHcRed1-C1: 0.48 ± 0.04, n. cells = 88; pSCAT3-DEVD + pHcRed1-C1-survivin: 0.27 ± 0.003, n. cells = 91; t-Test *P* = 0.014). We have then used a two-way ANOVA to measure simultaneously the effects of survivin overexpression and presence/absence of serum on ECFP_em_/Venus_em_, and to assess whether or not there was an interaction between these two parameters (four replicas). As expected, survivin overexpression was effective in reducing ECFP_em_/Venus_em_ (*P* = 1.00165E-05), whereas presence/absence of serum was not (*P* = 0.33), with no interaction of the two parameters (*P* = 0.17). Therefore, survivin overexpression reduced the level of Casp3 activation in CGCs independently of culture conditions.

We have then counted TCD of HcRed1 expressing-CGCs after transfection with pHcRed1-C1 or pHcRed1-C1-survivin in serum-free medium, and we found it increased of 2.82 fold in cells overexpressing survivin (Fig. 4f CTR bars - pSCAT3-DEVD + pHcRed1-C1: 5.0 ± 0.81, n. cells = 142; pSCAT3-DEVD + pHcRed1-C1-survivin: 14.09 ± 1.98, n. cells = 448; t-Test *P* = 0.010). From these observations we concluded that reduction of Casp3 activity in survivin overexpressing CGCs was paralleled by improved survival.

In keeping with our present observations, it was suggested that survivin, in addition to keeping alive the proliferating progenitors cells of central neurons during neurogenesis, also prevented the apoptotic death of neuronal precursors and post-mitotic neurons [20], such are CGCs in this study. In further support, survivin-defective mice demonstrated an increased activity of Casp3 and 9 in CNS [20, 41, 42]. Therefore, this group of experiments not only confirmed that Casp3 was constitutively active in CGCs and subjected to IAP negative modulation, but also that the apoptotic intracellular machinery was fully operational in transfected cells, which could be rescued from death by experimentally manipulating their survivin content, as well as by post-transcriptionally silencing the casp3 gene by RNAi.

### Quantification of HcRed1 fluorescence and its correlation with survivin expression and Casp3 activity

As we observed that the intensity of HcRed1 fluorescence was higher in cells overexpressing survivin than in control cells, we devised another set of experiments to establish whether or not survivin somehow interfered with plasmid transcription efficiency. In these experiments, we have measured the intensity of fluorescence (IF) emission (618 nm – arbitrary units) of the red fluorophore (IF_emHcRed1_) encoded by pHcRed1-C1 and pHcRed1-C1-survivin. IF_emHcRed1_ provides an indication of the level of transgene expression by the cell, as it is proportional to the amount of fluorochrome transcribed. Our observations demonstrated a statistically significant increase of IF_emHcRed1_ in survivin-overexpressing cells in comparison to controls (Fig. 4c - pHcRed1-C1: 35.78 ± 11.19; n. cells = 58; pHcRed1-C1-survivin: 102.57 ± 10.15, n. cells = 139; t-Test *P* = 0.0005). We then asked whether or not the differences in IF_emHcRed1_ were indeed correlated to different levels of activation of Casp3, rather than simply reflecting a better cellular state independent from survivin inhibition of the Casp3-mediated apoptotic drive. Therefore, we measured IF_emHcRed1_ and ECFP_em_/Venus_em_ in individual double-transfected CGCs (pHcRed1-C1, n. cells = 23; pHcRed1-C1-survivin, n. cells = 31). We observed a significant difference in both IF_emHcRed1_ and ECFP_em_/Venus_em_ between pHcRed1-C1 and pHcRed1-C1-survivin transfected cells [IF_emHcRed1_: 40.01 ± 4.45 (pHcRed1-C1) versus 69.93 ± 10.16 (pHcRed1-C1-survivin), t-Test: *P* = 0.010; ECFP_em_/Venus_em_: 0.17 ± 0.005 (pHcRed1-C1) versus 0.11 ± 0.007 (pHcRed1-C1-survivin), t-Test: *P* = 1.70132E-09]. In these experiments (Fig. 4d, e), we also noted that IF_emHcRed1_ in survivin-overexpressing cells was inversely proportional to ECFP_em_/Venus_em_ (R2 = 0.42 ± 0.07), whereas in cells transfected with the empty vector (pHcRed1-C1) no correlation was detectable (R2 = 0.0003 ± 0.03). Therefore, this set of observations further confirmed that survivin negatively modulated Casp3 activity in transfected CGCs.

Then, in the attempt to induce apoptosis, we employed H_2_O_2_ (from 50 μM up to25 mM) in different experimental settings. We have thus calculated TCD of HcRed1-expressing cells (Fig. 4f) as well as ECFP_em_/Venus_em_ (Fig. 4g) after double transfection with pSCAT3-DEVD + pHcRed1-C1 or pSCAT3-DEVD + pHcRed1-C1-survivin, and H_2_O_2_ challenge. In these experiments we did not find a significant difference between TCDs in control and H_2_O_2_-treated OCCs (5 mM – 48 h) after transfection with pHcRed1-C1 (*control:* 4.78 ± 0.81, n. cells = 539; *H*_*2*_*O*_*2*_*:* 4.25 ± 0.93, n. cells = 302; t-Test: *P* = 0.66). After transfection with pHcRed1-C1-survivin, TCD of HcRed1-expressing cells challenged with H_2_O_2_ was, instead, 46 % that in untreated controls (*control:* 14.09 ± 1.98, n. cells = 890); *H*_*2*_*O*_*2*_*:* 6.39 ± 2.19, n. cells = 203; t-Test: *P* = 0.020). On the other hand, the mean ECFP_em_/Venus_em_ values did not significantly change in cells transfected with pHcRed1-C1 (*control:* 0.32 ± 0.02, n. cells = 539; *H*_*2*_*O*_*2*_*:* 0.32 ± 0.009, n. cells = 302; t-Test: *P* = 0.89) or pHcRed1-C1-survivin (*control:* 0.24 ± 0.018, n. cells = 890; *H*_*2*_*O*_*2*_*:* 0.26 ± 0.018, n. cells = 203; t-Test: *P* = 0.67). We have performed similar experiments in pSCAT3-DEVD transfected cells after treatment with 25 mM H_2_O_2_ for twenty-four hours, and confirmed that H_2_O_2_ significantly reduced TCD without affecting the mean values of ECFP_em_/Venus_em_ (data not shown). Notably, at these peroxide concentrations, OCCs displayed clear morphological alterations (slice shrinkage, disintegration, and cellular swelling) that were indicative of a high degree of sufferance. In agreement with our findings, 50 μM H_2_O_2_ was demonstrated to activate Casp3 in Jurkat T-lymphocytes, but to induce Casp3-independent necrosis at higher concentrations up to 500 μM [43]. Authors have concluded that H_2_O_2_ had two distinct effects on cell death: it initially inhibited the caspases and delayed apoptosis. Then, depending on the degree of the initial oxidative stress, caspases were activated and death was apoptotic, or caspases remained inactive and necrosis occurred. Altogether, we interpreted this last group of findings as a further proof for the existence of a subpopulation of CGCs that were resistant to Casp3-mediated cell death: under basal conditions, Casp3 was active in these CGCs and they, being already committed to die, were apparently insensitive to oxidative stress; on the other hand, when cells were rescued from death by survivin they could be killed by oxidative stress, but without intervention of Casp3.

### Live imaging allows monitoring of Casp3 activity fluctuations under basal conditions and in response to pharmacological manipulation

As apoptosis of CGCs is known to be a very rapid phenomenon [16], we asked whether or not it was possible to monitor Casp3 activation in individual alive cells for an extended period of time (up to twenty-four hours - experiment #16 in Table 1).

**Fig. 5:**
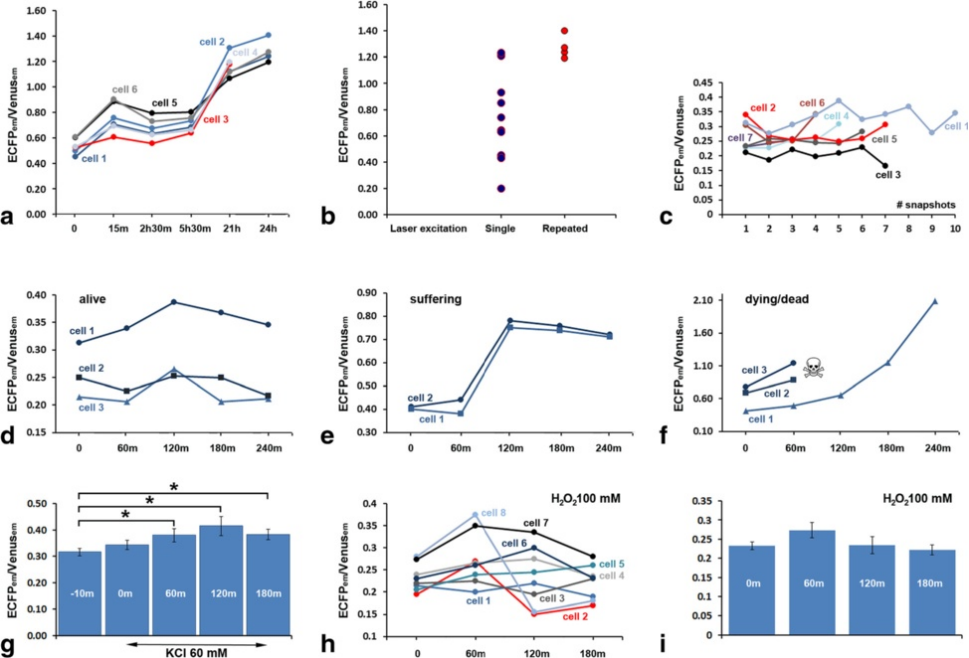
FRET measurements of Casp3 activity in live OCCs. **a**-**b** Repetitive imaging with a 40x lens results in an artifact increase of the ECFP_em_/Venus_em_ values recorded from the same cells in a 24-h interval. **a** progressive increase in the ECFP_em_/Venus_em_ ratio in six CGCs monitored at subsequent intervals up to 24 h. **b** comparison of the ECFP_em_/Venus_em_ ratios in two groups of cells undergoing single (*left - blue dots*) or repeated (*right - red dots*) laser excitation to capture FRET pair images. **c** Imaging with a 20x lens and repetitive laser excitations did not significantly alter the ECFP_em_/Venus_em_ ratio in seven CGCs that were photographed up to ten times to acquire FRET pair images. **d**-**f** Exemplificative traces of the fluctuations in the ECFP_em_/Venus_em_ ratio in healthy (**d**), suffering (**e**) or dying/dead (**f**) CGCs during a four-hour follow up. FRET was measured in these eleven cells during the course of the same experiment. Cell 2 and 3 in **f** died soon after one hour from start, and could not be subsequently identified when the culture was scanned for the acquisition of images at 120 m. It should be noted that live imaging can be made difficult by X-Y axis and focus (Z axis) drifting of the sample. If in-focus images cannot be captured at all time-points, cell(s) must be discharged from subsequent analysis. **g** = Effect of 60 mM KCl depolarization on the ECFP_em_/Venus_em_ ratio during a three-hour follow up (see text for further explanation). **h** Exemplificative traces of the fluctuations in the ECFP_em_/Venus_em_ ratio during a three-hour follow up in eight CGCs challenged with 100 mM H_2_O_2_. **i** Lack of a statistically significant response to 100 mM H_2_O_2_ in a subpopulation of CGCs that appeared to be resistant to oxidative stress (see text for discussion). Error bars = SEM. * *P*-value 0.05–0.01 %

Figure 5a shows the trend of the values of the ECFP_em_/Venus_em_ ratio in six CGCs that, while alive, were repeatedly photographed with a 40x lens to acquire image pairs for subsequent FRET analysis. Despite some fluctuations, we saw in all cells a progressive increase of ECFP_em_/Venus_em_, in parallel with the time of permanence in culture. Comparison of the ECFP_em_/Venus_em_ at beginning and end of experiment showed a statistically significant difference (t = 0: 0.55 ± 0.02; t = 24 h: 1.17 ± 0.04, n. cells = 6; t-Test *P* = 0.00005), and, at the end of observations, cells displayed more or less severe morphological changes indicative of apoptosis. To exclude that repeated laser excitations of ECFP at 435 nm was detrimental to cell survival, and, thus, we were recording a non-physiological artifact increase of Casp3 activity, we have measured ECFP_em_/Venus_em_ within a twenty-four hour interval in two different groups of cells. In this experiment, we collected FRET snapshots only at time 0 and 24 h in group 1 cells, whereas group 2 cells were subjected to a total of six progressive FRET measurements as above (Fig. 5b). These observations clearly demonstrated that the increase of Casp3 activity was an artifact. Therefore, we have devised another series of experiments by using a 20x lens. Positively, under these methodological conditions the ECFP_em_/Venus_em_ value displayed some degree of variability, but not a constant tendency to increase with time in parallel with multiple laser excitations (Fig. 5c).

Having proved with reasonable certainty that low magnification imaging did not significantly interfere with cell viability even after multiple laser exposures, we have employed the 20x lens for all subsequent observations.

These were first aimed to establishing if our approach could permit live cell visualization of NOND. As an example, Figure 5d-f show the fluctuations in ECFP_em_/Venus_em_ in CGCs that remained alive (Fig. 5d), displayed clear morphological signs of sufferance (Fig. 5e), or died (Fig. 5f) during a four-hour observation. Notably ECFP_em_/Venus_em_ remained well below 0.40 in all healthy cells, whereas it was already quite high at beginning of experiments in suffering (Fig. 5e) or dying cells (Fig. 5f, cells 2 and 3). The time course of the ECFP_em_/Venus_em_ in one of the dying cells (Fig. 5f cell 1) was particularly interesting, as this cell appeared to be healthy when first monitored, but reached a very high ECFP_em_/Venus_em_ during the last two hours of follow-up. We believe to have been fortunate enough to monitor in this cell the entire process of NOND, as the time-scale of the progressive increase in Casp3 activity was well coincident with the length of the process that we have calculated after labeling *in vivo* of proliferating/apoptotic CGCs in a previous study [44].

In the last two experiments, we have used real time live imaging to follow the response of CGCs to high depolarization (60 mM KCl – Fig. 5g) or strong oxidative stress (100 mM H_2_O_2_ – Fig. 5h-i). We here wanted to exclude that the experiments where cells were monitored only once, at the end of treatment and after fixation, were not fully suitable to capture a quick dynamics of response. We had taken into consideration this possibility because, as mentioned previously, when challenged with 60 mM KCl for 2 DIV in the course of transfection, no FRP-expressing cells were detectable in fixed OCCs. Not totally unexpectedly, we observed that CGCs responded to KCl with a statistically significant increase in the mean ECFP_em_/Venus_em_ starting from one hour after KCl challenge (Fig. 5g). In the subsequent two hours, the level of Casp3 activity remained significantly higher than at starting point, but did not significantly increased further. It is well know that neurons in slices respond to depolarizing concentrations of [K^+^]_e_ with an increase in [Ca^++^]_i_ that can be easily monitored by calcium imaging with LSCFM [45]. Therefore, the rise in ECFP_em_/Venus_em_ that followed 60 mM KCl was likely a Casp3 response to an altered intracellular Ca^++^ homeostasis. Notably, the values assumed by ECFP_em_/Venus_em_ during the exposure to 60 mM KCl were higher than those in physiological [K^+^]_e_ (5 mM) at beginning of observation, but not so high to induce cell death, at least in the time interval of our experiments. KCl-induced rise in [Ca^++^]_i_ promoted the activation of Casp3 and caused excessive apoptosis when rat hippocampal neurons were subjected to a toxic insult, but this was not the case in the absence of the toxicant [46]. Therefore, response of CGCs to sustained hyperpolarization indicated that these neurons have the capability, at least for a few hours, to adjust their cCasp3 levels to be eventually escaping death in OCCs.

In the last set of experiments we followed the response of CGCs to H_2_O_2_ oxidative stress during an interval of four hours. Figure 5h shows the fluctuations Casp3 activity in eight cells challenged with 100 mM H_2_O_2_. The mean values ECFP_em_/Venus_em_ at 0, 60, 120 and 180 min are reported in Fig. 5i. After statistical analysis, there were no significant differences at any of the time-points monitored in the course of the experiment. These observations excluded the possibility that a single FRET measurement of fixed OCCs was unfitted to capturing the quick dynamics of the apoptotic process, and further confirmed that a subpopulation of CGCs did not respond to oxidative stress with Casp3-mediated cell death.

### Technical advantages of the use of pSCAT3 to detect apoptosis *ex vivo*

Synthesis of the fusion protein containing the FRET pair and of other FRPs obviously needs well functional cellular machinery. This notion reinforces the importance of data gathered *ex vivo*, as it would be impossible acquiring similar information with extraction techniques such as Western blotting, or Casp3 enzyme assays. In addition, the sensitivity of Western blot is quite low, and blotting methods require obtaining protein samples from considerable numbers of cells [47]. Among histological techniques, the ICC localization of active cCasp3 is considered to be one of the most reliable methods to detect apoptosis in tissue sections, provided that specific antibodies are available. However, also this type of approach does not allow obtaining direct functional information on the enzyme activity, and, even most importantly, is not amenable to application in live cells.

Casp3 is the main executor protease in apoptosis and its activation was considered as a major irreversible end-step in cell death. Activation of Casp3 is indeed a late event in PCD, but it occurs well in advance prior to several biochemical and morphological changes of apoptosis [48]. Since the cascade of caspases’ activation is rapid and apoptotic cells are very quickly removed by microglia [49], the statistical probability to detect cCasp3 by ICC remains relatively low in the brain tissue during the course of NOND. Nonetheless, we and others have been able to localize Casp3 by ICC during post-natal cerebellar development in several species of altricial mammals, including humans [50]. However, NOND in cerebellum is characterized by massive apoptotic phenomena that occur along a relatively limited time-span: it was therefore not surprising that the number of cCasp3 immunoreactive cells detected in our previous studies was by far lower than that of successfully transfected CGCs in which Casp3 activity could be monitored here by pSCAT3-FRET.

In discussing the advantages of the pSCAT3-FRET approach to the study of NOND, it should be mentioned the possible alternative use of cell-penetrating peptide probes that can be activated by caspases. In general, probes constructed so far also use FRET, but consist of a non-fluorescent acceptor that, when Casp3 is inactive inside the cell, quenches the fluorescence of donor. The first of these probes to be developed consisted of a cell-penetrating Tat peptide conjugated to the effector recognition sequence DEVD flanked by a fluorophore (Alexa Fluor® 647)-quencher (QSY®21) pair [51]. Once exposed to the action of caspases, the recognition sequence is cut, resulting in separation of the fluorophore from the quencher and unmasking of the Alexa fluorescent signal. A second-generation probe, prepared by the same researchers, was called KcapQ, as it consisted of the recognition sequence KKKRKV, which is common to a group of effector caspases [13]. In HeLa cells treated with doxorubicin to induce apoptosis, KcapQ displayed increased sensitivity to caspases (Casp3 > 7 > 6), greater efficiency of the fluorophore-quencher pair, and lower cell toxicity compared to the first-generation molecule. Another probe of similar chemical features, TcapQ, was employed for the detection of retinal ganglion cells’ (RGCs) apoptosis after intravitreal injection of NMDA [12]. When TcapQ was injected before treatment with the agonist, it was possible to observe the fluorescent signal and its co-localization with TUNEL labeling of fragmented DNA. More recently, others developed a FRET pair consisting of Cy5 + QSY®21, and linked it with the brain-targeted peptide RVG29, so that the whole probe could cross the blood brain barrier and target the nervous tissue with high affinity [52]. Indeed the nano-device was functional and, after rotenone-induced apoptosis, accumulation of Cy5 fluorescence in several areas of the brain could be demonstrated. Even more recently, another Cy5/QSY®21-based probe (QCASP3.2) was developed and administered intravenously to successfully detect apoptotic cells after permanent focal ischemia in the mouse striatum [15].

Casp3 probes based on cell penetrating peptides are now commercially available in kit, to be used on isolated cells. We used one of these kits to monitor Casp3 activation in OCCs without success, as, rather than specific labeling of individual cells, widespread background fluorescence was observed, most likely because it was difficult for the reagent to effectively penetrate cerebellar slices in their thickness. The possibility to skip transfection and to deliver a probe with (minimally) invasive procedures is, of course, one major advantage of the methods based on the use of cell penetrating peptides. However, the case of RGCs was particular, as these cells and their axons are separated by the site of injection (the vitreous body) only by the thin inner limiting membrane of the retina. Obviously, the possibility to image probes that cross the blood brain barrier after intravenous administration has a great translational potential. However, the cellular resolution of these probes is, at the moment, far lower than that obtained here: it seemed difficult, at least from published images, to obtaining exact information on the type(s) of cells undergoing apoptosis, and to gather quantitative data at the single cell level. On the other hand, pSCAT3 was already demonstrated to be functional in embryos after *in ovo* transgenesis [53], and the possibility to use the GeneGun® approach *in vivo*, although technically demanding, is rather appealing to avoid the labor- and time-consuming procedure of generating new transgenic strains.

### Functional significance of Casp3 activation in CGCs

An excellent comprehensive discussion of the role of apoptosis in cerebellar development has very recently been published in the more general framework of resuming current knowledge on the commitment and fate of the cerebellar neurons [50]. In altricial mammals, CGCs are affected by two subsequent waves of apoptotic cell death during the course of their post-natal generation and migration [49]. The two waves, respectively, strike the neuronal progenitors (pre-migratory neuroblasts), and the mature post-migratory CGCs [16]. In the past, we have provided data as regarding the possibility that the apoptotic cell death of the CGC progenitors was Casp3-independent [11, 16]. Others have described an intervention of Casp3 during the course of CGC progenitor apoptosis, when OCCs were subjected to insulin-deprivation [54]. Notably, based on some discrepancies between the numbers of apoptotic cells after ICC or TUNEL, these authors hypothesized that at least some CGCs in their cultures died in a Casp3-independent manner.

The present study uses a totally new approach to dynamically analyze the site(s) of Casp3 activation at cellular and histological levels. Here we have shown that the enzyme is constitutively active in CGCs, and that activity can be observed at all stages of their differentiation and migration. We also demonstrated here that Casp3 activation does not simply follow an off-on regulation in live neurons: this implies that quantification of Casp3 activity is of primary importance for a correct understanding of the role of the protease in NOND and neuropathology, as the mere localization of cCasp3 might be confounding. Under this perspective, it is worth mentioning that we have previously demonstrated a post-translational upregulation of Bcl2, one of the most important mammalian anti-apoptotic proteins, as a response to extracellular/intracellular changes of K^+^ and/or Ca^++^ in CGCs [21]. Our present findings are in line with the possibility that CGCs, which will be successful in surviving NOND or in antagonizing a perturbation of their ionic milieu, dynamically counteract cleavage of Casp3 via, among others, a Bcl2-dependent mechanism.

However, the discovery that Casp3 displays a low level of activity in a subset CGCs, and that these cells are resistant to challenge with apoptosis inducers or oxidative stressor strengthens the notion that Casp3 in neurons may also promote non-apoptotic functions [9, 55]. It is thus useful recalling that we have been able to capture here the existence of spots of intense Casp3 activity in neuronal processes, at a developmental stage when synapses are formed in the cerebellar cortex. This observation is compatible with the very recent demonstration that the enzyme intervenes in the elimination of postsynaptic structures [56], and that its deficiency results in disrupted synaptic homeostasis [57].

## Conclusions

We have described here the implementation and refinement of a FRET-based methodology that is capable of capturing the functional and histological dynamics of Casp3 activation in individual mouse cerebellar neurons at a cellular level of resolution. Not only this approach has several advantages when compared to the currently existing methodologies for monitoring neuronal apoptosis, but it is amenable to be combined with the experimental manipulation of the apoptotic machinery inside the cell, eventually providing quantitative results that are fundamental for a comprehension of the role of Casp3 in live nerve cells. From the comparisons of the different sets of our experiments, the SCAT3 probes display remarkable uniformity in the readout of ECFP_em_/Venus_em_ under different physiological and experimental conditions such in basal constitutive activity (0.50–0.80), Casp3 RNAi, Ac-DEVD-CMK inhibition, survivin overexpression, resistance to apoptosis inductors (0.25–0.40), or oxidative stress-induced apoptosis (>0.80). Collectively, these observations prove that the probes are very stable and insensitive to changes in cellular environment that could otherwise result in changes of FRET_eff_ and, thence, ECFP_em_/Venus_em_. Our protocol *ex vivo* can be immediately applied to monitor the activity of the enzyme in slices from any areas of the brain and in retina, and has the potential to be transferred *in vivo*.

From the present study we provide a new hypothesis on the mechanism of regulation of Casp3 in cerebellum, and demonstrate the existence of a subpopulation of CGCs that does not respond to pro-apoptotic stimuli with increased Casp3 activity. As the role of Casp3 in the normal and pathological brain is gaining more and more importance, we believe that our approach is of relevance for a better comprehension of the regulation of NOND and molecular neurodegeneration in disease.

## Methods

### Ethics statement

All procedures were carried out according to regulations on experimental animal husbandry and care/welfare of the Italian Ministry of Health and University of Turin. They were approved by the Commission of Bioethics at the University of Turin and the Ministerial Office in charge for Protection of Experimental Animals. Animals were reared in the animal house of the Department of Veterinary Sciences, University of Turin. Housing conditions were those provided by the DL 26/2014 (protection of experimental animals). In addition, although not required by law, we kept all animals in an enriched environment by providing them with the living conditions laid down by the EU recommendations on the welfare of experimental animals (Directives 2010/63/EU of the European Parliament and 22/09/10 of the European Council on protection of animals bred for scientific purposes).

### Animal manipulation and collection of tissue samples

Mice (*n* = 86) at post-natal day 5 (P5) were euthanized with an overdose of sodium pentobarbital. The brain was quickly removed and placed in ice-cooled Gey’s solution (Sigma Chemicals, St. Louis, MO) supplemented with glucose and antioxidants (for 500 mL: 50 % glucose 4.8 mL, ascorbic acid 0.05 g, sodium pyruvate 0.1 g). The cerebellum was then isolated and immediately sectioned in 400 μm parasagittal slices with a McIlwain tissue chopper (Brinkmann Instruments, Westbury, NY), while submerged in a drop of cooled Gey’s solution.

### Preparation of OCCs

Three to five cerebellar slices were plated onto Millicell-CM inserts (Millipore, Billerica, MA). Each insert was subsequently placed inside a 35 mm Petri dish containing 1.1 mL of culture medium, and incubated at 34 ° C in 5 % CO_2_ for up to 8 days *in vitro* (DIV). Two different culture media (medium 1 - *with* horse serum; medium 2 - *without* horse serum) were employed in various sets of experiments (Table 1). Medium 1 was 50 % Eagle basal medium (BME, Sigma Chemicals), 25 % horse serum (Gibco®, Life Technologies™, Carlsbad, CA), 25 % Hanks balanced salt solution (HBSS, Sigma Chemicals), 0.5 % glucose, 0.5 % 200 mM L-glutamine, and 1 % antibiotic/antimycotic solution. Medium 2 was 95 % Neurobasal Medium A (Gibco®), 2 % B27 supplement 50X (Gibco®), 2 % 100 mM L-glutamine, and 1 % antibiotic/antimycotic solution. In all experiments, immediately after explant OCCs were plated in medium 1 and incubated for 4 DIV to allow them equilibrating to the *in vitro* conditions. On the fifth day, immediately after biolistic transfection, OCCs were transferred into fresh medium 1 or 2 according the experimental protocol. Further details on the procedure can be found in [21].

### Plasmids

The following plasmids have been used:

- *FRET probes*: FRET probes consisted of a functional probe (pSCAT3-DEVD) and a control probe (pSCAT3-DEVG). The two plasmids encode a FRET pair consisting of ECFP as a donor, and Venus (a mutant form of YFP) as an acceptor, linked by either the Casp3 recognition and cleavage sequence DEVD or the inappropriate sequence DEVG. In the latter, a glycine substituted for a critical aspartic acid as the fourth residue of cleavage site renders it insensitive to Casp3 activity. Coding is under the control of the hCMV promoter. Excitation/emission maxima are 435 nm/475 nm (ECFP) and 475 nm/530 nm (Venus) [24, 58].
- *Survivin probes*: pHcRed1-C1, pHcRed (Clontech Laboratories, Mountain View, CA), and pHcRed1-C1-survivin encode for HcRed1. The protein emits in the far red, with excitation/emission maxima at 588 nm/618 nm. pHcRed1-C1-survivin was obtained after insertion of the DNA sequence coding for survivin in the MCS, fused with the C-terminal sequence of HcRed1 [59]. HcRed1 maintains its characteristics of fluorescence even when used as a fusion protein and acts, therefore, as a reporter protein allowing the localization of successfully transfected cells by means of red fluorescence emission.
- *shRNA plasmids*: Casp3 RNAi experiments were performed using a commercially available kit (Casp3 SureSilencing shRNA Plasmids, Quiagen, Hilden, Germany). The kit contains four different shRNA plasmids and a negative control plasmid. Plasmids also encode GFP as a reporter protein.

Plasmid DNAs were amplified and harvested from competent *E. coli* cultures following conventional procedures, and suspended in TE buffer at a stock concentration of 1 μg DNA/μL for downstream applications.

### Biolistic transfection

The basic protocol for biolistic transfection was modified from [21]. All procedures were carried out at room temperature, unless otherwise stated. Gold particles (1 μm) were coated with plasmid DNA as follows: in a microfuge tube, 500 μL of a 50 mg/mL suspension of gold particles (DNAdel^TM^ Gold Carrier Particles – S1000d, Seashell Technology, LLC, La Jolla, CA) were added to 330 μL binding buffer (Seashell Technology) upon vortexing, to a final concentration of 30 mg gold/mL. After sonication, 50 μg of plasmid DNA (single transfection), or 25 + 25 μg of two different plasmid DNAs (double transfection) were added, so that the gold-to-DNA ratio (50/25 = 2 μg/mg) remained unchanged. While vortexing, an equal volume of precipitation buffer was added (380 μL for 1 μg/μL DNA stocks), and the tube was left to stand for 3 min. In RNAi experiments, gold particles were coated with the four different shRNA plasmids or with the shRNA control plasmid together with pSCAT3-DEVD or pSCAT3-DEVG. In these experiments, we employed a slightly higher gold-to-DNA ratio (2.5 μg/mg) to have more gold available for coating. The gold-to-DNA ratio was maintained constant in all different types of cartridges (Additional file 5).

The DNA-gold complex was then spun at 10,000-x *g* in a microfuge for 10 s to pellet the DNA-coated gold particles. After removal of the supernatant the pellet was first resuspended in 500 μL of cold ethanol, then vortexed, pelleted, and again resuspended in 3.5 mL cold ethanol. The gold suspension was briefly sonicated, transferred into the Tubing Prep Station® (Bio-Rad, Hercules, CA), and cartridges were prepared according to the manufacturer’s protocol. Slices were transfected by the Helios Gene Gun® (Bio-Rad) at an operating pressure of 160 psi and by placing the barrel liner over the target at a distance of 1.6 mm with a spacer. One single shot was given to each culture dish. Additional details on the procedure can be found in [60].

### ICC

To identify transfected CGCs and to localize cCasp3, OCCs shot with the Helios Gene Gun® were processed for ICC as described elsewhere [29]. The rabbit polyclonal antibody against NeuN (Abcam, Cambridge, UK) was used at a dilution of 1:1,500. The rabbit polyclonal antibody against the large fragment (17/19 kDa) of activated Casp3 does not recognize full length Casp3 or other cleaved caspases (#9661 Cell Signaling Technology, Danvers, MA). It was used at a dilution of 1:200.

### Live imaging

A custom-made incubation chamber was used for live imaging LSCFM of OCCs (Tokai Hit, Fujinomiya-shi, Japan). The chamber was described in details elsewhere [60]. It was mounted over the stage of a SP5 LSCFM (Leica Microsystems, Wetzlar, GE), so that the OCCs grown on the Millicell-CM insert membrane could be directly observed at different time intervals and medium replaced as necessary without moving the insert from the stage. This allowed the recognition of individual cells in serial snapshots from the same microscopic field in long-term follow up experiments.

### Monitoring caspase-3 activation by FRET

#### Calculation of FRET efficiency (FRET_eff_) of the pSCAT3 probe

FRET_eff_ was calculated after acceptor photobleaching, by measuring the resultant increase in donor fluorescence after destroying the acceptor [61]. The procedure was carried out by comparing donor fluorescence intensity in the same cells *before* and *after* destroying the acceptor by full-power laser excitation. After transfection with pSCAT3-DEVG, OCCs were fixed (see below) and individual cells subjected to bleaching by repeated excitement of the Venus fluorochrome with the 514 nm laser at maximum power and a 63x oil lens (NA = 1.4). The energy transfer efficiency was quantified as:

$$ FRE{T}_{eff} = \left({\mathrm{D}}_{\mathrm{post}}-{\mathrm{D}}_{pre}\right)/{\mathrm{D}}_{\mathrm{post}} $$

Where

**D**_post_ = fluorescence emission intensity of the donor *after* acceptor photobleaching; **D**_**pre**_ = fluorescence emission intensity of the donor *before* acceptor photobleaching; ***FRET***_***eff***_ is positive when **D**_**post**_ > **D**_**pre**_. For further information see Additional file 1.

### Fixed OCCs

Forty-eight hours after transfection, OCCs were fixed in 4 % paraformaldehyde (PFA) in 0.1 M phosphate buffer (pH 7.4–7.6) for one hour at room temperature [29]. In [52], for simultaneous detection of pSCAT3 ratio changes and Casp3 activation by ICC, SCAT3^+^ transgenic embryos were fixed with 2 PFA and 0.1 % glutaraldehyde in phosphate-buffered saline for 20 min on ice. We decided to avoid glutaraldehyde fixation as it gives high auto-fluorescence, besides to having a detrimental effect on antigenicity, and increased the % of PFA for better morphological preservation. After several washes in buffer, we mounted OCCs on glass slides and observed them with the confocal microscope. For FRET detection, the 458 nm excitation line from the argon ion laser at 30 % power was used to excite ECFP. Images were taken by the use of a 63x oil lens (NA = 1.4) or a 40x dry lens (NA = 1) with appropriate ECFP and Venus filters. The emission filter sets used were: band-pass BP470–500 nm, for ECFP, and long-pass LP530 nm for Venus. A region of interest (ROI) corresponding to the entire cell body was drawn for each CGC, and two separate 1024x1024 images of the same field were captured, one for each of the two FRET fluorophores. All microscope settings remained unchanged during capture. In dual labeling experiments, a third 1024x1024 image was acquired for the red channel, to record HcRed1 fluorescence (λ = 630 nm). See Additional file 4 for further details on this procedure, and Additional file 3 for the procedure used to measure FRET after excitation of ECFP with a 405 nm laser line.

### Living OCCs

To measure FRET in living OCCs, 4 DIV cultures transfected with probe-containing plasmids were transferred into the incubation chamber mounted on the SP5 stage. We subjected OCCs to pharmacological treatments (see Table 1) and monitored them at different time intervals up to twenty-four hours. At each monitoring point, different sets of digital images were acquired according to the experimental paradigm employed (two images for single transfection experiments, three for double transfections), as described above. Images were taken with a 40x water immersion lens (NA = 0.8), or a 20x dry lens (NA = 0.5).

### Statistical analysis

We performed statistical analysis on raw data, using the Excel spreadsheet Analysis Tool Pack. Student’s t-Test for independent groups was used when two conditions at a time were matched after F-test for equality of two variances. ANOVA was applied to compare data divided in more than two groups. We reported data as means ± SEM, with n. indicating the number of cells. For each experimental condition, we recorded cells from at least three different OCCs. We considered statistically significant *P* values ≤0.05.

### Calculation of TCD

We calculated TCD with the Image J software (https://imagej.nih.gov/ij/). First, we photographed each cerebellar slice in OCCs with a 5x lens under a transmitted/fluorescence light microscope (DM6000B, Leica) equipped with appropriate filter combinations to detect GFP, Venus or HcRed1. Then, we calculated the total area of each slice, applied a threshold to isolate fluorescent cells from the underlying background, and finally used the Count Objects command to calculate the number and areas of transfected cells. Measurements were set to take into consideration only objects with areas falling into the 20–314 μ^2^ range. This corresponded to hypothetic circular cells of 5–20 μM in diameter.

### Drugs

A23187 (calcimycin), H_2_O_2_, NMDA, and KA were from Sigma Chemicals. Caspase-3 inhibitor III (Ac-DEVD-CMK) was from Santa Cruz Biotechnology, Dallas TX.

## Additional files

Additional file 1: Calculation of FRET efficiency by acceptor photobleaching. Protocol for acceptor photobleaching – Data on acceptor photobleaching. (DOCX 29 kb) Additional file 2: Main advantages/disadvantages of the methods available for monitoring FRET_eff_ at single cell level. Comparative tables and references for FRET protocols. (DOCX 40 kb) Additional file 3: Measurement of FRET in RNAi experiments using the 405 nm laser line. Measurement of FRET after excitation of CYFP at 405 nm to show that FRET can be successfully measured in multiple transfected cells expressing GFP as a reporter protein for shRNA transfection. An image of a group of multiple transfected cells is provided. (DOCX 2651 kb) Additional file 4: Measurement of FRET in double transfected CGCs. Examples of the measurement of the ECFP_em_/Venus_em_ ratio in six pSCAT3-DEVD + pHcRed1-C1 double transfected cells. (DOCX 1342 kb) Additional file 5: Preparation of cartridges for Casp3 RNAi experiments. Protocol to prepare multiple plasmid DNA-coated gold particles for biolistic experiments. (DOCX 18 kb)

## Abbreviations

cCasp3: cleaved Casp3
CGCs: cerebellar granule cells
DsRed1: wild-type Discosoma red fluorescent protein
ECFP: enhanced cyan fluorescent protein
E-FRET: sensitized acceptor fluorescence
EYFP: enhanced yellow fluorescent protein
FLIM-FRET: donor fluorescence lifetime
FRET: fluorescence resonance energy transfer
FRPs: fluorescent reporter proteins
hCMV: human cytomegalovirus
HcRed1: humanized DsRed1
HPT: hours post-transfection
IAPs: inhibitors of apoptosis proteins
ICC: immunocytochemistry
LSCFM: laser scanning confocal fluorescence microscopy
NA: numerical aperture
NMDA: N-methyl-D-aspartate
NOND: naturally occurring neuronal death
OCCs: organotypic cerebellar cultures
p: plasmid
PCD: programmed cell death
RGCs: retinal ganglion cells
RNAi: RNA interference
ROI: region of interest
sh: short hairpin
TCD: transfected cell density
