# Supplementary material for: Ex vivo imaging of active caspase 3 by a FRET-based molecular probe demonstrates the cellular dynamics and localization of the protease in cerebellar granule cells and its regulation by the apoptosis-inhibiting protein survivin
Source: Mol Neurodegener. 2016 Apr 28;11:34. doi: 10.1186/s13024-016-0101-8 (PMC4848850; doi:10.1186/s13024-016-0101-8)
Supplement: Additional file 1: — Calculation of FRET efficiency by acceptor photobleaching. Protocol for acceptor photobleaching – Data on acceptor photobleaching. (DOCX 29 kb) [file 13024_2016_101_MOESM1_ESM.docx]

**Calculation of FRET efficiency by acceptor photobleaching**

***Acceptor photobleaching protocol***

Acquire snapshot 63X images from an OCC mounted in PBS-glycerol 1:9

| **CAPTURE IMAGE** | **BLEACHING** | **CAPTURE IMAGE** |
| --- | --- | --- |
| 1. 400Hz 2. 1024x1024 3. laser 458nm 30% power | 1. 400Hz 2. 1024x1024 3. laser 514nm 100% power 4. zoom 8X | 1. 400Hz 2. 1024x1024 3. laser 458nm 30% power |

***Measurements***

| **Cells #** | **ECFP_pre_** | **ECFP_post_** | **Venus_pre_** | **Venus_post_** |
| --- | --- | --- | --- | --- |
| **1** | 18.27 | 23.77 | 31.96 | 19.95 |
| **2** | 15.07 | 19.29 | 25.15 | 15.24 |
| **3** | 30.25 | 35.33 | 57.78 | 38.39 |
| **4** | 43.42 | 57.34 | 81.54 | 41.07 |
| **5** | 50.23 | 65.92 | 63.78 | 42.22 |
| **6** | 45.72 | 71.2 | 91.06 | 50.55 |
| **7** | 46.16 | 61.06 | 60.7 | 47.79 |
| **8** | 59.29 | 69.01 | 73.92 | 51.86 |
| **9** | 44.71 | 53.67 | 60.59 | 46.85 |
| **10** | 33.71 | 44.27 | 146.66 | 68.17 |
| **11** | 33.77 | 47.94 | 125.37 | 70.17 |
| **12** | 13.9 | 15.3 | 46.93 | 36.56 |

| **Cells #** | **ECFP_pre_/Venus_pre_** | **ECFP_post_/Venus_post_** | **% BLEACH** | **FRET_eff_** |
| --- | --- | --- | --- | --- |
| **1** | 0.57 | 1.19 | 62.42 | 0.23 |
| **2** | 0.60 | 1.27 | 60.60 | 0.22 |
| **3** | 0.52 | 0.92 | 66.44 | 0.14 |
| **4** | 0.53 | 1.40 | 50.37 | 0.24 |
| **5** | 0.79 | 1.56 | 66.20 | 0.24 |
| **6** | 0.50 | 1.41 | 55.51 | 0.36 |
| **7** | 0.76 | 1.28 | 78.73 | 0.24 |
| **8** | 0.80 | 1.33 | 70.16 | 0.14 |
| **9** | 0.74 | 1.15 | 77.32 | 0.17 |
| **10** | 0.23 | 0.65 | 46.48 | 0.24 |
| **11** | 0.27 | 0.68 | 55.97 | 0.30 |
| **12** | 0.30 | 0.42 | 77.90 | 0.09 |
| **Mean** | 0.55 | 1.10 | 64.01 | 0.22 |
| ***s*** | 0.20 | 0.36 |  | 0.07 |
| **SEM** | 0.06 | 0.10 |  | 0.02 |

***Statistics***

| F-Test Two-sample for Variances |  |  |
| --- | --- | --- |
|  |  |  |
|  | *Post-bleach* | *Pre-bleach* |
| Mean | 1.104046608 | 0.551032191 |
| Variance | 0.126522028 | 0.041039478 |
| Observations | 12 | 12 |
| df | 11 | 11 |
| F | 3.082934643 |  |
| P(F<=f) one-tail | 0.037441153 |  |
| F critical one-tail | 5.319667079 |  |
|  |  |  |

| t-Test: Two Sample Assuming Unequal Variances |  |  |
| --- | --- | --- |
|  |  |  |
|  | *Pre-bleach* | *Post-bleach* |
| Mean | 0.551032191 | 1.104046608 |
| Variance | 0.041039478 | 0.126522028 |
| Observations | 12 | 12 |
| Hypothesized Mean Difference | 0 |  |
| df | 17 |  |
| t Stat | -4.679936377 |  |
| P(T<=t) one-tail | 0.000107589 |  |
| t Critical one-tail | 2.89823052 |  |
| P(T<=t) two-tail | 0.000215178 |  |
| t Critical two-tail | 3.222449911 |  |
