# Supplementary material for: Ex vivo imaging of active caspase 3 by a FRET-based molecular probe demonstrates the cellular dynamics and localization of the protease in cerebellar granule cells and its regulation by the apoptosis-inhibiting protein survivin
Source: Mol Neurodegener. 2016 Apr 28;11:34. doi: 10.1186/s13024-016-0101-8 (PMC4848850; doi:10.1186/s13024-016-0101-8)
Supplement: Additional file 2: — Main advantages/disadvantages of the methods available for monitoring FRETeff at single cell level. Comparative tables and references for FRET protocols. (DOCX 40 kb) [file 13024_2016_101_MOESM2_ESM.docx]

**Main advantages/disadvantages of the methods available for monitoring FRET_eff_ at single cell level**

|  |  | **Advantages** | **References** |
| --- | --- | --- | --- |
|  |  | **Disadvantages** |  |
| **Methods** | E-FRET | - Very precise quantification of molecular interactions | [1] |
|  |  | - Multiple sets of images are required - Donor-only and acceptor-only reference samples are required - Impracticable for live-cell imaging |  |
|  | FLIM-FRET | - Quantification of molecular interactions | [2] |
|  |  | - Very expensive - Specific instrumentation required - Prone to methodological errors |  |
|  | Quenching of donor fluorescence | - Relatively easy to perform - Applicable to live cell imaging | [3,4] |
|  |  | - Complete photobleaching may be difficult - Damage to alive cells may be serious |  |

*Abbreviations*: E-FRET = sensitized acceptor fluorescence; FLIM-FRET = donor fluorescence lifetime

**Comparisons of FRET_eff_ measurements with SCAT3 and other FRET probes**

|  |  | **FRET pair** | **Main features**  **Advantages/Disadvantages** | **Theoretical FRET_eff_** | **References for isolated cells** |
| --- | --- | --- | --- | --- | --- |
| **Probes** | SCAT3 | ECFP/Venus | - Fluorochrome stoichiometric ratio 1:1 - 18-aa linker - Insensitive to changes in cellular ionic concentrations | 0.35±0.08  (17-aa linker) [5]  0.22±0.02 (this work) | [6,7] |
|  | EYFP-based | ECFP/EYFP | - Changes in intracellular concentration of H^+^ or Cl^-^ may cause FRET to detect artifact signals |  | [8-10] |

References

1. Hoppe A, Christensen K, Swanson JA. **Fluorescence resonance energy transfer-based stoichiometry in living cells.** Biophys J 2002;**83**:3652-64.

2. Hoffmann B, Zimmer T, Klocker N, Kelbauskas L, Konig K, Benndorf K, et al. **Prolonged irradiation of enhanced cyan fluorescent protein or Cerulean can invalidate Forster resonance energy transfer measurements.** J Biomed Opt 2008;**13**:031205.

3. Pelet S, Previte MJ, Kim D, Kim KH, Su TT, So PT. **Frequency domain lifetime and spectral imaging microscopy.** Microsc Res Tech 2006;**69**:861-74.

4. Gu Y, Di WL, Kelsell DP, Zicha D. **Quantitative fluorescence resonance energy transfer (FRET) measurement with acceptor photobleaching and spectral unmixing.** J Microsc 2004;**215**:162-73.

5. Koushik SV, Chen H, Thaler C, Puhl HL, III, Vogel SS. **Cerulean, Venus, and VenusY67C FRET reference standards.** Biophys J 2006;**91**:L99-L101.

6. Wu Y, Xing D, Luo S, Tang Y, Chen Q. **Detection of caspase-3 activation in single cells by fluorescence resonance energy transfer during photodynamic therapy induced apoptosis.** Cancer Lett 2006;**235**:239-47.

7. Wang L, Chen T, Qu J, Wei X. **Quantitative analysis of caspase-3 activation by fitting fluorescence emission spectra in living cells.** Micron 2009;**40**:811-20.

8. Tyas L, Brophy VA, Pope A, Rivett AJ, Tavare JM. **Rapid caspase-3 activation during apoptosis revealed using fluorescence-resonance energy transfer.** EMBO Rep 2000;**1**:266-70.

9. Luo KQ, Yu VC, Pu Y, Chang DC. **Application of the fluorescence resonance energy transfer method for studying the dynamics of caspase-3 activation during UV-induced apoptosis in living HeLa cells.** Biochem Biophys Res Commun 2001;**283**:1054-60.

10. Rehm M, Dussmann H, Janicke RU, Tavare JM, Kogel D, Prehn JH. **Single-cell fluorescence resonance energy transfer analysis demonstrates that caspase activation during apoptosis is a rapid process. Role of caspase-3.** J Biol Chem 2002;**277**:24506-14.
