# Supplementary material for: Ex vivo imaging of active caspase 3 by a FRET-based molecular probe demonstrates the cellular dynamics and localization of the protease in cerebellar granule cells and its regulation by the apoptosis-inhibiting protein survivin
Source: Mol Neurodegener. 2016 Apr 28;11:34. doi: 10.1186/s13024-016-0101-8 (PMC4848850; doi:10.1186/s13024-016-0101-8)
Supplement: Additional file 3: — Measurement of FRET in RNAi experiments using the 405 nm laser line. Measurement of FRET after excitation of CYFP at 405 nm to show that FRET can be successfully measured in multiple transfected cells expressing GFP as a reporter protein for shRNA transfection. An image of a group of multiple transfected cells is provided. (DOCX 2651 kb) [file 13024_2016_101_MOESM3_ESM.docx]

**Measurement of FRET in RNAi experiments using the 405 nm laser line**

In RNAi experiments transfected cells express the FRET pair and GFP (figure below). As excitation cross talk may occur when ECFP is excited at 458 nm (see Fig. 4a in [1]), FRET was also measured after excitation at 405 nm. Results confirmed that expression of GFP in multiple transfected cells did not preclude measurement of FRET, and that RNAi significantly lowered the ECFP_em_/Venus_em_ from 0.57 to 0.43 (see Statistics) also under these experimental conditions.

***Statistics***

| F-Test Two-sample for Variances |  |  |  |
| --- | --- | --- | --- |
|  |  |  |  |
|  | *DEVD* | *DEVD siRNA* |  |
| Mean | 0.578938392 | 0.430748212 |  |
| Variance | 0.038177553 | 0.027691325 |  |
| Observations | 48 | 59 |  |
| df | 47 | 58 |  |
| F | 1.378682745 |  |  |
| P(F<=f) one-tail | 0.121968953 |  |  |
| F critical one-tail | 1.575145029 |  |  |
|  |  |  |  |
| t-Test: Two Sample Assuming Equal Variances | |  |  |
|  | |  |  |
|  | | *DEVD* | *DEVD siRNA* |
| Mean | | 0.578938392 | 0.430748212 |
| Variance | | 0.038177553 | 0.027691325 |
| Observations | | 48 | 59 |
| Pooled variance | | 0.03238516 |  |
| Hypothesized Mean Difference | | 0 |  |
| df | | 105 |  |
| t Stat | | 4.236440743 |  |
| P(T<=t) one-tail | | 2.44413E-05 |  |
| t Critical one-tail | | 1.659495383 |  |
| P(T<=t) two-tail | | 4.88825E-05 |  |
| t Critical two-tail | | 1.982815274 |  |

Exemplificative images of a group of CGCs multiple transfected with pSCAT3-DEVD and the mix of the four shRNA plasmids to silence caspase 3. ShRNA plasmids also encode GFP as a reported protein. The three images show in pseudocolor the fluorochromes introduced into the cells by biolistic transfection. Note that all cells express the three FRP that can be selectively excited at different wavelengths. The possibility to easily perform multiple transfections is one of the main advantages of the biolistic procedure. *Abbreviations:* ECFP = enhanced cyan fluorescent protein; GFP = green fluorescent protein; Venus = Venus variant of the enhanced yellow fluorescent protein; shRNAi = short hairpin plasmid for RNA interference. Scale bars = 10 µm.

Reference

1. Broussard JA, Rappaz B, Webb DJ, Brown CM. **Fluorescence resonance energy transfer microscopy as demonstrated by measuring the activation of the serine/threonine kinase Akt.** Nat Protoc 2013;**8**:265-81.
