# Supplementary material for: Ex vivo imaging of active caspase 3 by a FRET-based molecular probe demonstrates the cellular dynamics and localization of the protease in cerebellar granule cells and its regulation by the apoptosis-inhibiting protein survivin
Source: Mol Neurodegener. 2016 Apr 28;11:34. doi: 10.1186/s13024-016-0101-8 (PMC4848850; doi:10.1186/s13024-016-0101-8)
Supplement: Additional file 4: — Measurement of FRET in double transfected CGCs. Examples of the measurement of the ECFPem/Venusem ratio in six pSCAT3-DEVD + pHcRed1-C1 double transfected cells. (DOCX 1342 kb) [file 13024_2016_101_MOESM4_ESM.docx]

**Measurement of FRET in double transfected CGCs**

| **Cell #** | *Fluorescence intensity (arbitrary units)* | | | |
| --- | --- | --- | --- | --- |
|  | **475 nm** | **530 nm** | **ECFPem/Venusem** | **618 nm** |
| 1 | 6.94 | 153.33 | 0.05 | 144.17 |
| 2 | 11.17 | 143.59 | 0.08 | 84.29 |
| 3 | 21.84 | 172.04 | 0.13 | 54.61 |
| 4 | 19.31 | 146.49 | 0.13 | 38.47 |
| 5 | 21.81 | 141.81 | 0.15 | 31.89 |
| 6 | 13.67 | 68.93 | 0.20 | 8.40 |

Example of the measurement of the ECFP_em_/Venus_em_ ratio in six double transfected cells (pSCAT3-DEVD+pHcRed1-C1). ROIs are indicated by the white circles. The fluorescence intensity is calculated as the mean of fluorescence emission in ROI using the FRET Wizard software of the SP5 confocal microscope.
