# Supplementary material for: Ex vivo imaging of active caspase 3 by a FRET-based molecular probe demonstrates the cellular dynamics and localization of the protease in cerebellar granule cells and its regulation by the apoptosis-inhibiting protein survivin
Source: Mol Neurodegener. 2016 Apr 28;11:34. doi: 10.1186/s13024-016-0101-8 (PMC4848850; doi:10.1186/s13024-016-0101-8)
Supplement: Additional file 5: — Preparation of cartridges for Casp3 RNAi experiments. Protocol to prepare multiple plasmid DNA-coated gold particles for biolistic experiments. (DOCX 18 kb) [file 13024_2016_101_MOESM5_ESM.docx]

**Preparation of cartridges for Casp3 RNAi experiments**

Multiple DNAs were adsorbed onto carrier gold particles in the preparation of cartridges for Casp3 RNAi experiments according to the Table below:

| **Cartridges** | **Gold (mg)** | **FRET plasmids (μg)** | | **shRNA plasmids (μg)** | | | | |
| --- | --- | --- | --- | --- | --- | --- | --- | --- |
|  |  | **DEVD** | **DEVG** | **Contr Clone** | **Clone 1** | **Clone 2** | **Clone 3** | **Clone 4** |
| **DEVD** | **25** | **62.5** |  |  |  |  |  |  |
| **DEVD/RNAi** | **25** | **62.5** |  |  | **15.6** | **15.6** | **15.6** | **15.6** |
| **DEVD/RNAiCONTR** | **25** | **62.5** |  | **62.5** |  |  |  |  |
| **DEVG** | **25** |  | **62.5** |  |  |  |  |  |
| **DEVG/RNAi** | **25** |  | **62.5** |  | **15.6** | **15.6** | **15.6** | **15.6** |
| **DEVG/RNAiCONTR** | **25** |  | **62.5** | **62.5** |  |  |  |  |

The gold-to-DNA ration was set at 2.5 m to have a larger gold surface available for DNA adsorption – for discussion see [67]. The total amount of plasmidic DNA for shRNA clones 1-4 was set to the same value than that of the plasmidic DNA of FRET probes (15.6 x 4 = 62.4 and 62.5 respectively).
